# Global warming and arctic terns: Estimating climate change impacts on the world's longest migration

**DOI:** 10.1111/gcb.16891

**Published:** 2023-07-26

**Authors:** Joanne M. Morten, Pearse J. Buchanan, C. Egevang, Isolde A. Glissenaar, Sara M. Maxwell, Nicole Parr, James A. Screen, Freydís Vigfúsdóttir, Noam S. Vogt‐Vincent, Daniel A. Williams, Ned C. Williams, Matthew J. Witt, Lucy A. Hawkes, William Thurston

**Affiliations:** ^1^ Department of Biosciences, Faculty of Health and Life Sciences University of Exeter, Hatherly Laboratories Exeter UK; ^2^ Department of Earth, Ocean and Ecological Sciences University of Liverpool Liverpool UK; ^3^ Greenland Institute of Natural Resources Nuuk Greenland; ^4^ Bristol Glaciology Centre, School of Geographical Sciences University of Bristol Bristol UK; ^5^ School of Interdisciplinary Arts & Sciences University of Washington Bothell Washington USA; ^6^ Department of Mathematics and Statistics, Faculty of Environment, Science and Economy University of Exeter Exeter UK; ^7^ School of Social Sciences University of Iceland Reykjavík Iceland; ^8^ Department of Earth Sciences University of Oxford Oxford UK; ^9^ Met Office Exeter UK

**Keywords:** arctic tern, climate change, CMIP6, migration, net primary productivity, sea ice, *Sterna paradisaea*, wind

## Abstract

Climate change is one of the top three global threats to seabirds, particularly species that visit polar regions. Arctic terns migrate between both polar regions annually and rely on productive marine areas to forage, on sea ice for rest and foraging, and prevailing winds during flight. Here, we report 21st‐century trends in environmental variables affecting arctic terns at key locations along their Atlantic/Indian Ocean migratory flyway during the non‐breeding seasons, identified through tracking data. End‐of‐century climate change projections were derived from Earth System Models and multi‐model means calculated in two Shared Socioeconomic Pathways: ‘middle‐of‐the‐road’ and ‘fossil‐fuelled development’ scenarios. Declines in North Atlantic primary production emerge as a major impact to arctic terns likely to affect their foraging during the 21st century under a ‘fossil‐fuelled development’ scenario. Minimal changes are, however, projected at three other key regions visited by arctic terns (Benguela Upwelling, Subantarctic Indian Ocean and the Southern Ocean). Southern Ocean sea ice extent is likely to decline, but the magnitude of change and potential impacts on tern survival are uncertain. Small changes (<1 m s^−1^) in winds are projected in both scenarios, but with minimal likely impacts on migration routes and duration. However, Southern Ocean westerlies are likely to strengthen and contract closer to the continent, which may require arctic terns to shift routes or flight strategies. Overall, we find minor effects of climate change on the migration of arctic terns, with the exception of poorer foraging in the North Atlantic. However, given that arctic terns travel over huge spatial scales and live for decades, they integrate minor changes in conditions along their migration routes such that the sum effect may be greater than the parts. Meeting carbon emission targets is vital to slow these end‐of‐century climatic changes and minimise extinction risk for a suite of polar species.

## Graphical abstract

Climate change is a major threat to seabirds. Through their reliance on both polar regions, arctic terns could be particularly vulnerable. Twenty‐first century trends in environmental variables during arctic tern migration and non‐breeding seasons are reported. By 2100, primary production in the North Atlantic is likely to decline, whereas the magnitude of decline in Southern Ocean sea ice extent is uncertain. Projected changes to winds are unlikely to impact arctic tern migration. Although arctic terns appear resilient to these projected changes, there could be compounding impacts of multiple small changes. Meeting emission targets remains vital to permit species the space to adapt.

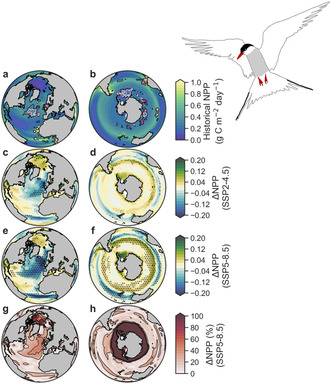

## 1 INTRODUCTION

Anthropogenic‐induced climate change is a grave concern to the survival of flora and fauna species globally (IPCC, 2022). Local species losses and extinction are predicted for one in six species under the fossil‐fuelled development climate scenario (Urban, 2015) and 15%–37% of species are predicted to be ‘committed to extinction’ by 2050 (Thomas et al., 2004). The speed and severity of climate change is more rapid in polar ecosystems than anywhere else on Earth (e.g. Previdi et al., 2021; Screen & Simmonds, 2010), with range expansion not possible for many polar species, and genotypic adaptations likely too slow (Convey & Peck, 2019; Gilg et al., 2012; Post et al., 2019). Animals that migrate to polar regions are also likely to encounter phenological mismatches with favourable weather or prey availability at one or more stages during their annual cycle (Carey, 2009). The rapidity of change combined with a susceptibility to phenological mismatch makes polar migrants especially vulnerable (Kubelka et al., 2022).

Most seabird species migrate to breed in temperate and polar regions (Hamer et al., 2002; Lascelles et al., 2014), relying on terrestrial habitats to breed, and marine habitats during the rest of their annual cycle. As such, they are vulnerable to a multitude of threats both on land and at sea, with climate change considered one of the top three threats globally (Dias et al., 2019). Seabirds are the most threatened bird group, with 31% of species classified as Vulnerable, Endangered or Critically Endangered by the IUCN (Dias et al., 2019), and as ocean sentinels they reflect ocean health (Furness & Camphuysen, 1997). One of the most iconic seabirds is the arctic tern (*Sterna paradisaea*, Pontoppidan, 1763), a small (*c*. 100 g) seabird that makes the longest annual migration of any animal on Earth (more than 100,000 km) between the Arctic during the boreal summer breeding season and the Antarctic during the boreal winter (Egevang et al., 2010). Covering vast parts of the planet, arctic terns are an ideal umbrella species to investigate the impacts of climate change on a suite of other marine species using the same regions. In the present study, we investigate how future climate change may impact arctic terns in breeding and non‐breeding regions, and along one major flyway through the Atlantic and Indian Oceans. We particularly focus on three key variables: net primary productivity (NPP) at key migratory stopover sites, sea ice extent in the Southern Ocean and prevailing wind conditions during migration and overwintering.

Marine NPP describes the rate of organic matter produced by phytoplankton (Heinsch et al., 2006). As the basis for almost all food chains, NPP can be used to infer abundance of heterotrophic species such as euphausiids (krill), which in turn are a keystone zooplankton prey species in many marine ecosystems, linking the phytoplankton on which they graze to higher trophic levels (Antezana, 2010; Montes‐Hugo et al., 2009; Pillar et al., 1992; Trathan & Hill, 2016). During arctic tern southbound migration, flight speeds tend to decrease in areas with high NPP levels, suggesting opportunistic feeding en route (Hensz, 2015). Thus, understanding how climate change may alter the rate and distribution of NPP is vital to anticipate food web reorganisations that affect predators like arctic terns. Key regions visited by arctic terns that follow the Atlantic flyway include the North Atlantic, the Benguela upwelling of southwest Africa, Amsterdam Island in the Indian Subantarctic and the Atlantic sector of the Southern Ocean (Alerstam et al., 2019; Egevang et al., 2010; Fijn et al., 2013). There are already changes to NPP recorded in these key regions. For example, multi‐decadal declines in NPP in the North Atlantic (that are highly likely to continue; Bindoff et al., 2019) are tightly correlated with declines in zooplankton abundance (Edwards et al., 2021) and losses in fish recruitment (Capuzzo et al., 2018).

Sea ice is a major driver of marine ecological processes in Antarctica, directly and indirectly affecting organisms from microscopic phytoplankton (Lin et al., 2021) to blue whales (*Balaenoptera musculus*, Linnaeus, 1758; Shabangu et al., 2020). During their non‐breeding season from November to February/March, arctic terns associate with the Antarctic pack‐ice zone (Alerstam, 1985; Redfern & Bevan, 2020; Salmonsen, 1967), concentrated in East Antarctica, the Weddell Sea, the Ross Sea and the Amundsen Sea (Gudmundsson et al., 1992). Arctic terns likely remain within the fragmented sea ice zone throughout the non‐breeding period due to high abundances of Antarctic krill (*Euphausia superba*, Dana 1850; Redfern & Bevan, 2020), and because ice provides a platform for rest during their annual moult (Alerstam et al., 2019; Cline et al., 1969). Projected declines in Antarctic krill spawning habitat of up to 80% by the end of the century, as a result of dramatic sea ice loss and altered seasonality (Pinkerton & Hayward, 2021), could therefore have significant effects on the global population of arctic terns.

Wind speed and direction can directly impact seabirds by influencing flight costs, with seabirds often using cross‐ and tailwinds to their advantage to fly cheaply at high ground speeds (Weimerskirch et al., 2000) and over long distances (Ventura et al., 2020). Wind conditions can strongly affect migration routes (e.g. Alerstam, 1979; Liechti, 2006). Shearwaters, for example, adjust their migratory routes to optimise wind conditions experienced rather than to reduce migration distance (González‐Solís et al., 2009), and in headwind conditions, seabirds are more likely to choose to fly closer to the coast in calmer conditions (Mateos & Arroyo, 2011). Seabirds also tend to fly at higher altitudes in stronger winds, though this varies with wing morphology and flight strategy (i.e. soaring, gliding, flapping; Ainley et al., 2015). For example, soaring albatross likely benefit from high wind speeds (Weimerskirch et al., 2000), whereas auks, which do not soar or glide, likely incur greater energetic costs (Elliott et al., 2014). Arctic terns likely benefit from wind support during their northbound migration, resulting in a faster journey than their southbound migration (Hromádková et al., 2020). Globally, average wind speeds have already increased, and extreme events have increased even more rapidly (Young et al., 2011). Regional changes (increases or decreases) of greater than 2% are predicted in most areas due to climate change (McInnes et al., 2011). In particular, coastal upwelling regions that support productive marine ecosystems have experienced intensified alongshore winds as a result of warming (Bakun et al., 2010; Sydeman et al., 2014). Under future climate change it is therefore possible that seabirds may need to alter their migratory or foraging routes, and that migration times could change, which could impact energetic costs.

In the present study, we investigate how changes to NPP, Southern Ocean sea ice and wind patterns under two Shared Socioeconomic Pathways (SSPs; Riahi et al., 2017) might impact: (i) foraging at key arctic tern stopover areas during southbound migration in the Atlantic flyway; (ii) arctic tern movements in the Southern Ocean; (iii) sea ice associated foraging areas in the Southern Ocean and (iv) the route and duration of their northbound migration (particularly with respect to tailwind support). We use tracking data supplemented with new datasets to identify key migration routes and foraging areas. In addition, we also examine how well wind patterns explain the routes taken by arctic terns.

## 2 METHODS

### 2.1 Tern tracking data

Tracking datasets were collected for a total of 21 arctic terns that migrated through the Atlantic and Indian Oceans, two with Global Positioning System tracking devices (deployed in Iceland in 2018 and scheduled to record a location every 18 h with an accuracy of a few metres, see Morten et al., 2022), and a further 19 terns with light geolocator devices (10 in Greenland, one in Iceland, see Egevang et al. (2010); and eight in Sweden, see Alerstam et al. (2019); https://doi.org/10.5061/dryad.d6080nt). Geolocator devices produce estimates of location with varying accuracy, but typically thought to be approximately 185 km (Lisovski et al., 2012; Rakhimberdiev et al., 2017), once or twice per day, with gaps during equinoxes or periods when the arctic terns were in 24 h of daylight. Important areas for arctic terns were identified by binning locations into regular 1° × 1° grid cells, the total time spent in each grid cell calculated, and a gaussian smoother applied.

### 2.2 Climate and environmental variables

Models were used to investigate changes experienced by arctic terns in vertically integrated net primary production (*intpp*), sea ice concentration (*siconc*) and near‐surface winds (in zonal and meridional directions; variables *uas* and *vas*). These variables were extracted from 36 Earth System Models (ESMs) listed in Table 1, and where possible, models that contained all variables were used. Historical and future trends were modelled assuming conditions in the SSPs developed for Phase 6 of the Coupled Modelling Intercomparison Project (CMIP6; Riahi et al., 2017). Historical simulations were driven by observed changes in greenhouse gas emissions, aerosols, ozone and land‐use changes from 1850 to 2014. Two SSP scenarios were used to explore future changes from 2015 to 2100: the high emissions or ‘fossil‐fuelled development’ scenario (SSP5‐8.5) and the moderate emissions or ‘middle‐of‐the‐road’ scenario (SSP2‐4.5). All model output was converted to a standard 1 × 1° (longitude by latitude) horizontal grid for analysis. Modelling took place over the full spatial extent over which arctic terns are known to breed, migrate and spend the non‐breeding season, and consequently extends outside the spatial range of tracking data compiled in the present study.

**TABLE 1:** CMIP6 source model data used within the analyses of this study (for both hind and fore casting). All data were interpolated and regridded to a 1° × 1° resolution on a latitude–longitude grid for comparison.

Centre	Model	Variables	Variant	Notes
AWI	AWI‐CM‐1‐1‐MR	*uas*, *vas*	r1i1p1f1	a ^,^ b
BCC	CSM2‐MR	*sico*	r1i1p1f1	
CAMS	CSM1‐0	*sico*	r1i1p1f1	
CAS	FGOALS‐f3‐L	*sico*	r1i1p1f1	
CCCma	CanESM5	*sico*, *uas*, *vas*	r1i1p1f1	a
CCCma	CanESM5	*intpp*	r1i1p2f1	
CMCC	CMCC‐ESM2	*uas*, *vas*	r1i1p1f1	a
CNRM‐CERFACS	CNRM‐CM6	*sico*, *uas*, *vas*	r1i1p1f2	b
CNRM‐CERFACS	CNRM‐CM6‐1‐HR	*sico*	r1i1p1f2	
CNRM‐CERFACS	CNRM‐ESM2‐1	*intpp*, *sico*, *uas*, *vas*	r1i1p1f2	a
CSIRO	ACCESS‐ESM1.5	*intpp*, *sico*, *uas*, *vas*	r1i1p1f1	a ^,^ b
CSIRO‐ARCCSS	ACCESS‐CM2	*sico*, *uas*, *vas*	r1i1p1f1	b
EC‐Earth	EC‐Earth3	*sico*	r1i1p1f1	
EC‐Earth	EC‐Earth3‐Veg	*sico*	r1i1p1f1	
FIO	FIO‐ESM‐2‐0	*sico*	r1i1p1f1	
GFDL	GFDL‐CM4	*sico*	r1i1p1f1	
GFDL	GFDL‐ESM4	*sico*	r1i1p1f1	
INM	INM‐CM4‐8	*sico*	r1i1p1f1	
INM	INM‐CM5‐0	*sico*	r1i1p1f1	
IPSL	ISPL‐CM6A‐LR	*intpp*, *sico*, *uas*, *vas*	r1i1p1f1	a ^,^ b
MIROC	MIROC‐ES2L	*intpp*, *sico*, *uas*, *vas*	r1i1p1f2	a
MIROC	MIROC6	*sico*, *uas*, *vas*	r1i1p1f1	b
MOHC	HadGEM3‐GC31‐LL	*sico*, *uas*, *vas*	r1i1p1f3	a
MOHC	HadGEM3‐GC31‐MM	*sico*	r1i1p1f3	c
MOHC	UKESM1.0‐LL	*intpp*, *sico*, *uas*, *vas*	r1i1p1f2	a
MPI‐M	MPI‐ESM1‐2‐HR	*intpp*, *sico*, *uas*, *vas*	r1i1p1f1	a ^,^ b
MPI‐M	MPI‐ESM1‐2‐LR	*sico*, *uas*, *vas*	r1i1p1f1	b
MRI	MRI‐ESM2‐0	*intpp*	r1i2p1f1	
MRI	MRI‐ESM2‐0	*sico*, *uas*, *vas*	r1i1p1f1	b
NCAR	CESM2	*intpp*	r4i1p1f1	
NCAR	CESM2‐WACCM	*sico*	r1i1p1f1	
NCC	NorESM2‐LM	*intpp*, *sico*	r1i1p1f1	
NCC	NorESM2‐MM	*sico*	r1i1p1f1	
NOAA‐GFDL	GFDL‐CM4	*intpp*, *uas*, *vas*	r1i1p1f1	a
NOAA‐GFDL	GFDL‐ESM4	*intpp*, *uas*, *vas*	r1i1p1f1	a
NUIST	NESM3	*sico*, *uas*, *vas*	r1i1:p1f1	b

^a^ *uas*, *vas* used in analysis for *Wind*.

^b^ *uas*, *vas* used in analysis for *vTern simulations*.

^c^ *sico* not available in this model for scenario SSP2‐4.5.

### 2.3 Net primary productivity

**FIGURE 1:**
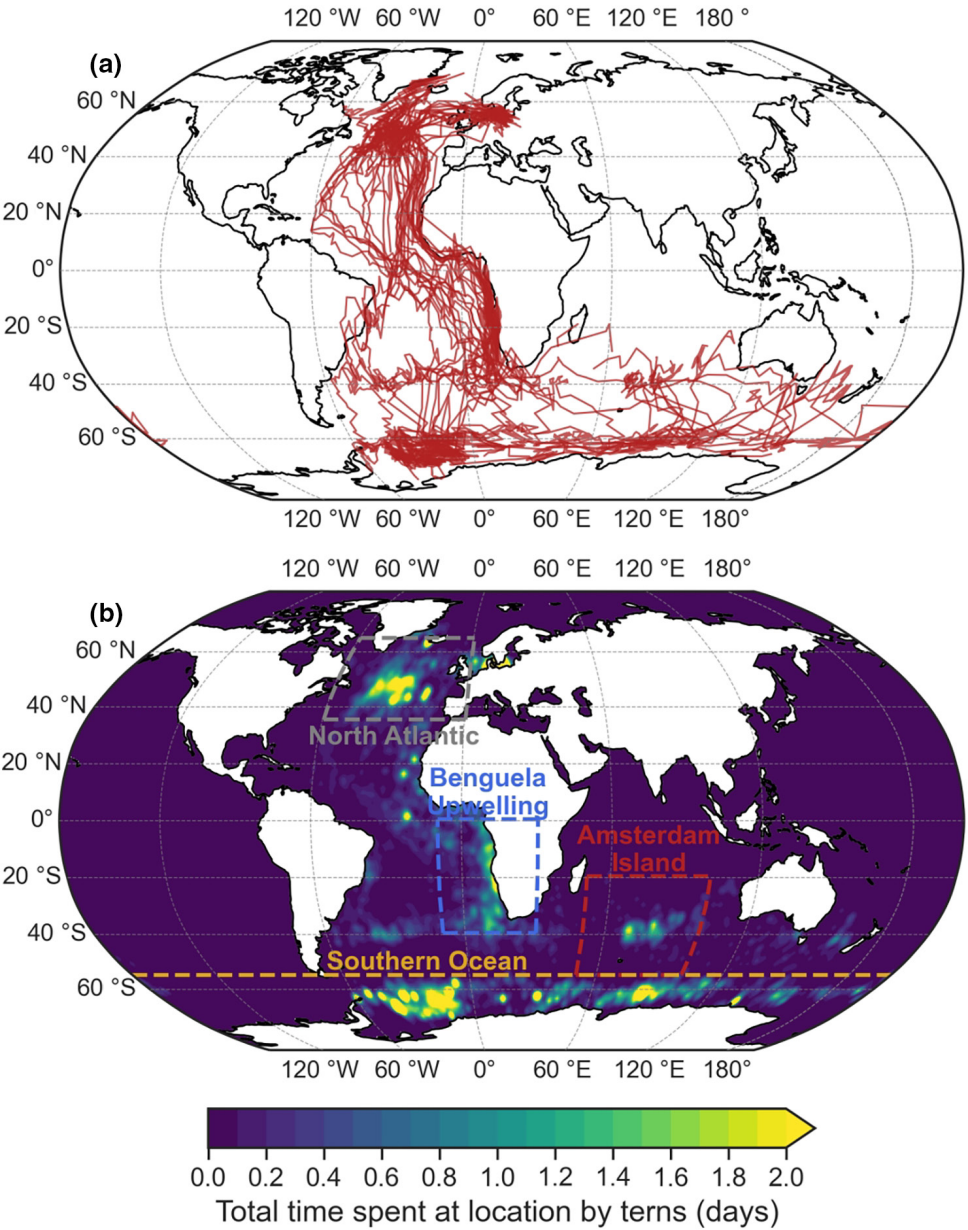
Maps showing (a) 23 migratory routes (red lines) recorded from 21 arctic terns tracked from 2007 to 2018. Note that lines connect successive locations, which may be spaced several days apart and can therefore result in the appearance of straight lines which are unlikely to have been the route taken; (b) Key foraging stopover and overwinter regions (highlighted in green to yellow showing increasing time spent at each location with boxes defining the extent of each).

Arctic terns stopover to refuel during their migration (McKnight et al., 2013) and visit several important areas (e.g. Davies, Carneiro, Tarzia, et al., 2021; Heerah et al., 2019). Four important stop‐over areas were selected based on the number of days arctic terns in the dataset were present in 1° × 1° horizontal grid cells, along with stopover sites suggested in published studies: (i) the North Atlantic subpolar gyre (between 40° N and 65° N and 60° W–0°; Egevang et al., 2010), (ii) the Benguela eastern boundary upwelling system (between 0°–40° S and 10° W and 30° E; Alerstam et al., 2019), (iii) Amsterdam Island in the Subantarctic Indian Ocean (between 20° S–55° S and 50° E–100° E; Fijn et al., 2013) and (iv) the seasonal sea ice zone in the greater Southern Ocean (latitudes poleward of 55° S; Redfern & Bevan, 2020). Within each region, we weighted NPP based on the number of tern days per grid, thereby focusing only on the stopover sites used by arctic terns (Figure 1b). Projections of vertically integrated marine NPP at these four areas were determined by 12 ESMs (Table 1), and the multi‐model mean changes were calculated for the present day (1995–2014) and future (2081–2100) relative to the preindustrial climate (1850–1950). NPP is used here as a proxy to indicate the quality of foraging conditions arctic terns encounter during their migratory and overwintering periods.

To assess whether there was a significant change in NPP at each of the four stop‐over areas, a signal to noise ratio approach was used (Hawkins & Sutton, 2012). Trends in NPP were calculated for each 1° × 1° grid cell using the multi‐model mean. Multi‐model mean NPP was then weighted by arctic tern density in each grid cell to return the mean and standard deviations (SD) of NPP for each stop‐over area. A significant change in NPP occurred when the long‐term trends in NPP (the signal) was greater than the inter‐annual variation (the noise, defined as one SD). We considered two approaches: one sensitive and the other conservative. Our sensitive approach considered that ‘normal’ conditions for arctic terns were those between 1850 and 1950 (i.e. near‐preindustrial conditions). The signal of change was calculated by subtracting mean NPP (weighted by arctic tern use) during the preindustrial period from the decadally smoothed trend in NPP between 1850 and 2100. If the signal in a given year ${\left\{\mathrm{NPP}\right\}}_{\text{year}}$ exceeded the envelope of normal conditions, then the signal to noise ratio (S:N) would exceed +1 or be less than −1 (Equation 1). In this case, the NPP and by extension foraging during that year was considered anomalous. Note that the S:N values could be positive or negative depending on the direction of the trend in NPP.
(1)

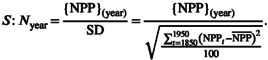




In our conservative approach, we considered the possibility that conditions in 1850–1950 were not necessarily optimal for arctic terns. Instead, we considered that ‘normal’ NPP conditions reflected those at the start of each successive generation of arctic terns, implying that newly fledged terns could ‘adapt’ to the new conditions within their lifetime (Equation 2; Figure S1). Arctic terns live for up to 34 years (Hatch, 2002), with an average adult lifespan of 11–19 years (Petersen et al., 2020). An arctic tern that fledged in 2000 might experience and adapt to conditions that are different to an arctic tern that fledged in 2050. In this case, the speed of environmental change (the signal) compared to environmental variability (the noise) over the lifetime of the bird is relevant. If change is slow but variability is high, terns may alter their behaviour to maximise fitness in these conditions, even if the climate is distinct from previous generations. On the other hand, if change is fast but variability is low, conditions will be significantly different at the end of their lifetime than compared to the beginning, and it is possible that birds of that generation will struggle to adapt. To estimate the signal experienced by a bird born in 2000 that lives for 15 years, for example, we calculated the linear trend in NPP from 2000 to 2014 (Figure S1a,b). To estimate the noise, we calculated the standard deviation of NPP over the same period (Figure S1c). If the signal of NPP exceeded the envelope of one SD around the mean, we considered that the signal was anomalous to the noise (S:N >1 or <−1). Again, signal to noise values could be negative or positive depending on whether NPP declines or increases at a given location (Figure S1d).
(2)

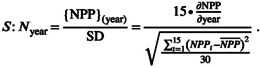




### 2.4 Sea ice

Arctic terns are present in the Southern Ocean during the austral summer (October–March). We examined the sea ice concentration and extent (defined as the contour in which sea ice concentration was 15% or greater) across the full longitudinal range of the Southern Ocean during those months. Historical projections of sea ice concentration data (from 1901 to 2014) and future projections (2015–2100) under the SSP2‐4.5 and SSP5‐8.5 scenarios were obtained from the 29 CMIP6 models (Table 1), and the decadal mean extents were calculated. Hindcast sea ice extents were compared to the observational data to check the model validity. Sea ice observations were accessed from the National Oceanic and Atmospheric Administration (NOAA) Optimal Interpolation (OI) v2 dataset (https://psl.noaa.gov/data/gridded/data.noaa.oisst.v2.html; Reynolds et al., 2002) and were available from December 1981 to April 2021. The product uses adjusted satellite passive microwave‐derived sea ice data from the NASA Goddard Team algorithm, followed by operational sea ice data from the National Centers for Environmental Prediction (NCEP). The observed decadal mean sea ice edge was calculated for each austral summer month (from 1982 to 2020). Recorded arctic tern locations in the austral summer of 2007/2008 were visually compared to the average monthly observed sea ice extent to determine how closely they associated with the sea ice edge.

During the austral summer (October–March), which includes the months that arctic terns were known to be present in the Southern Ocean and foraging at the sea ice edge (November–March), we supplemented observed sea ice concentrations with model‐derived estimates of marine vertically integrated NPP produced by the global ocean biogeochemistry hindcast simulation (https://resources.marine.copernicus.eu/product‐detail/GLOBAL_MULTIYEAR_BGC_001_029/INFORMATION). This ocean biogeochemistry model is forced by historically accurate atmospheric conditions, and thus discrepancies between observed and modelled sea ice concentrations are minimal. Remotely sensed chlorophyll estimates were not used because of (i) the significant underestimation of in situ chlorophyll by satellite algorithms in the Southern Ocean (Pope et al., 2017), (ii) the curtailed availability of chlorophyll data (1998–present) relative to sea ice data (1982–present), (iii) patchiness of data due to cloud cover that aliases monthly means and (iv) subsurface phytoplankton blooms not captured by satellite (Baldry et al., 2020).

### 2.5 Wind

Historical and future wind conditions were constructed from monthly averages of 12 models (see Table 1). Data for each model were resampled at 1° × 1° spatial resolution, and the multi‐model mean calculated for both *uas* and *vas*. Historical data were averaged over 1960–2014, and the projected future scenarios were averaged over the two emissions scenarios (SSP2‐4.5 and SSP5‐8.5) for 2080–2099, yielding three multi‐model datasets. Further monthly temporal averages were constructed for the three datasets, corresponding with the times when arctic terns would be present during their trans‐equatorial migration, and overwintering period in the Southern Ocean.

Wind conditions at high latitudes of the northern Atlantic were averaged between May and August, matching the arctic tern breeding season (Vigfúsdóttir et al., 2013). Conditions in the mid‐Atlantic and Indian Oceans were extracted from August to October, corresponding to the southbound migratory routes of arctic terns breeding in Europe and Eastern North America (e.g. Egevang et al., 2010; Wong et al., 2021). Similarly, data covering Southern Ocean and Antarctic regions were extracted from November to March, corresponding to the wintering season of arctic terns (e.g. Redfern & Bevan, 2020). Wind conditions were extracted from April to May in the Atlantic Ocean corresponding with the more rapid (e.g. Alerstam et al., 2019; Egevang et al., 2010; Fijn et al., 2013) northbound migration of arctic terns.

### 2.6 Virtual arctic terns

To model how the path and timing of arctic tern migration might change in the future, simple simulations were set up in Parcels v2.3 (Delandmeter & van Sebille, 2019; Lange & van Sebille, 2017), representing terns as virtual particles (i.e. virtual terns, henceforth ‘vTerns’). Arctic tern southbound migration was not simulated due to the route complexity, which includes multiple stopovers (e.g. Egevang et al., 2010) and ‘decisions’ such as how long to remain at a stopover site, which route to follow (the west African coast or eastern coast of South America), and at which latitude to enter the Southern ocean (this ranges from 20° E below the African continent to 160° E below the Tasman Sea). By contrast, the northbound migration of arctic terns tracked to date within the Atlantic flyway starts from the Weddell Sea and follows the same general shape through the central Atlantic. Additionally, the northbound migration is shorter (e.g. Hromádková et al., 2020) and therefore likely has less opportunity to mitigate future changes that could impact the duration of migration. vTern migration began at 70 °S in the Weddell Sea region, at equally spaced locations between 60° W and 40° W, which approximates the region from which arctic terns actually begin their northward migration (Figure 1). During active hours (defined as the first 14 h, or 60% of each day), vTerns were assumed to remain at low altitude. The mean flight altitude recorded during migration of the two arctic terns carrying GPS tracking devices was 88.7 m (±300.9 SD; Figure S2), and at low altitudes they remain within sight of the sea surface and therefore prey; (Hedenström & Åkesson, 2016). vTerns were (i) advected by the daily 10 m‐wind velocity and (ii) ‘flew’ at a constant airspeed of 10 m s^−1^ (Gudmundsson et al., 1992; Hedenström & Åkesson, 2016). vTerns were assumed to always fly towards Iceland (65° N, 20° W; a geographical central region between the Greenland, Icelandic and Swedish colonies of the tracked terns used here) during active hours, but the actual trajectories are not great circles as the vTerns can be pushed off course by winds. During inactive hours (defined as the last 10 h, or 40% of each day), vTerns were assumed to remain static, and were neither flying nor advected by wind. Once vTerns passed 60° N, they were removed from the simulation. These are the minimal assumptions required to reproduce the main features of the arctic tern northbound migration. Although more complex behavioural assumptions may further improve the ability of vTerns to reproduce modern migration paths, these behaviours may change in response to climate change. As a result, this minimal approach allows us to infer changes to the energetic cost of the northbound migration.

vTerns were released on the 80th, 90th and 100th days of the year (corresponding to the observed timing of the onset of the northbound migration: Alerstam et al., 2019; Egevang et al., 2010; Fijn et al., 2013), with 2000 vTerns generated on each release day, totalling 6000 vTerns annually. Releases occurred annually from 1850 (when available) or 1950 (otherwise) to 2014 (using the Historical scenarios), and from 2015 to 2099 (using SSP2‐4.5 and SSP5‐8.5 scenarios). Simulations were carried out using the 10 m‐wind velocity from 10 CMIP6 models (see Table 1). In each simulation, the position of each vTern was recorded every 2 h, as well as the total time taken to travel from 70° S to 60° N. A time‐step of 1 h was used for simulating vTern trajectories, which is the temporal resolution used to predict vTern travel time.

### 2.7 Data sources

CMIP6 models (see Table 1 for details) were accessed through JASMIN during a hackathon event that ran from 2 to 4 June 2021 and was organised by the University of Bristol and Cabot Institute (Mitchell et al., 2022). All CMIP6 model output is freely available on the Earth System Grid Federation (https://esgf.llnl.gov/), while the global ocean biogeochemistry hindcast simulations are available on the Copernicus Marine Database (https://resources.marine.copernicus.eu/).

## 3 RESULTS

### 3.1 Net primary productivity

**FIGURE 2:**
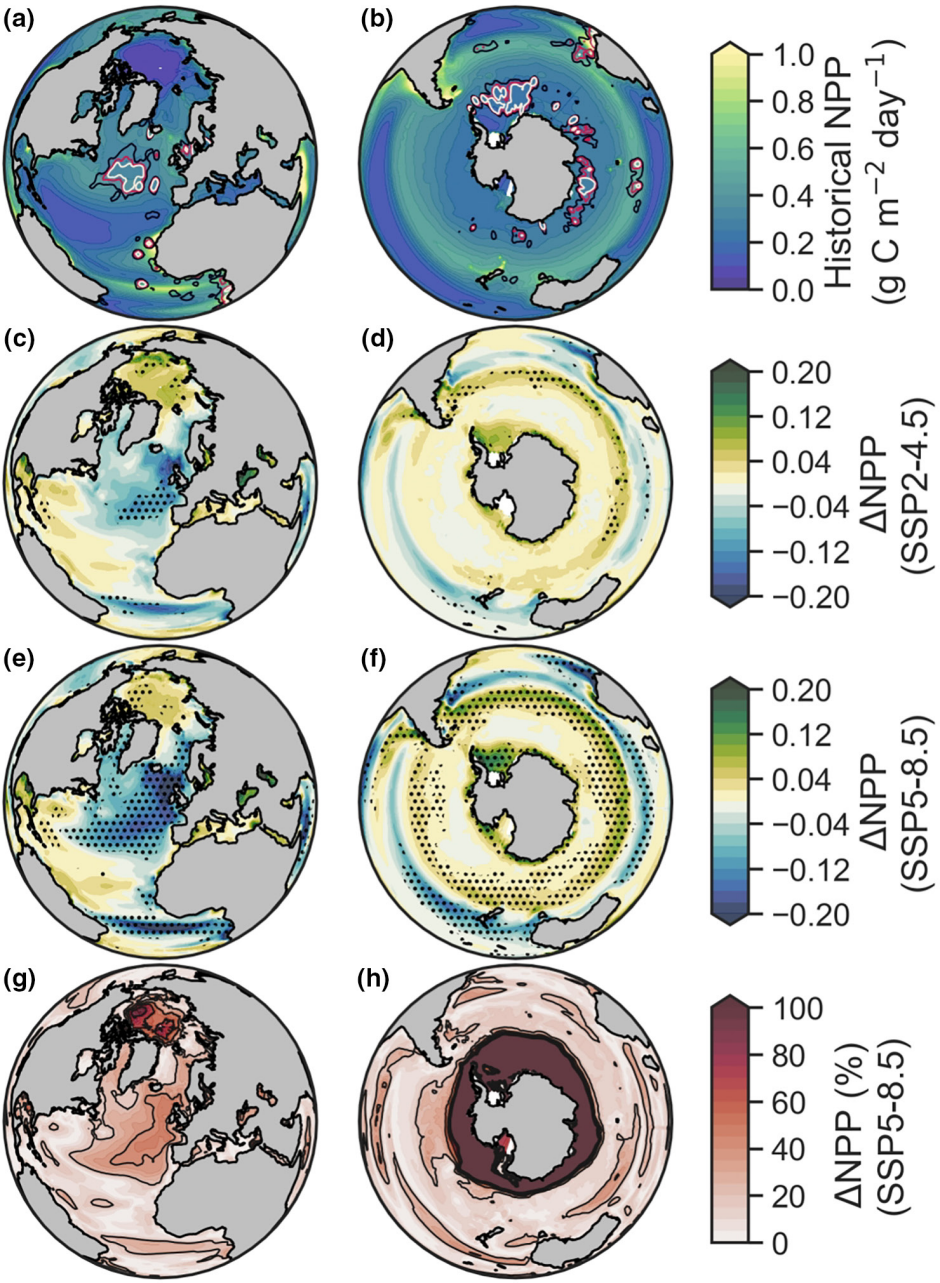
Multi‐model mean marine net primary production (NPP) projections. The northern hemisphere (left) shows average conditions in July–September and the southern hemisphere (right) shows average conditions in January–March. (a, b) Vertically integrated NPP under preindustrial conditions (1850–1950). Contours show high intensity foraging areas derived from Figure 1 (the contours equal 0.5 (white), 1.0 (red) and 1.5 (black) days of arctic tern presence). (c, d) Change in NPP (ΔNPP) at polar regions for the end of the 21st century (2081–2100) relative to the preindustrial for SSP2‐4.5. (e, f) As c and d, but for SSP5‐8.5. Hatching in panels c–f shows where at least 9 of 12 models (75%) agree on the direction of change. (g, h) The percentage change in NPP relative to preindustrial conditions at the end of the 21st century (2081–2100) for climate change scenario SSP5‐8.5. Contours are every 20%.

**FIGURE 3:**
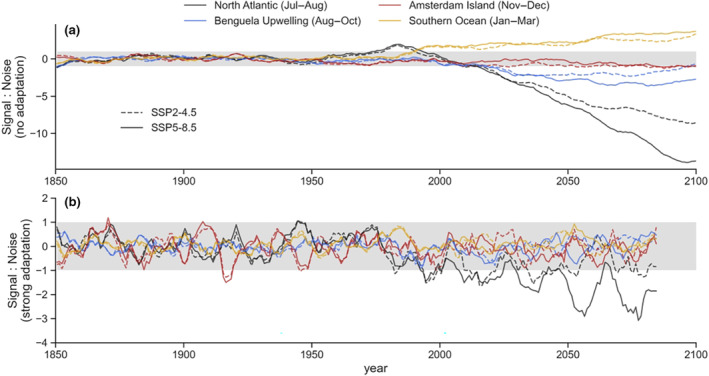
Trends in marine net primary production (NPP) and their statistical anomalies. (a) Signal to noise ratio in each major foraging region, where the climate‐driven trends (i.e. signals) are referenced to the 1850–1950 period. Values greater than 1 indicate an anomalous positive trend relative to the 1850–1950 period. Values less than 1 indicate an anomalous negative trend relative to the 1850–1950 period. Values between −1 and 1 indicate typical NPP relative to 1850–1950 (grey shading). If we assume no ability for arctic terns to adapt to new conditions, then values outside the envelope of −1 to 1 should affect the population. (b) Conservative signal to noise ratio in each major foraging region, where linear climate‐driven trends (i.e. signals) during a 15‐year period are referenced to the noise (SD) within that period. This analysis assumes each successive generation is able to fully adapt to new conditions during their lifetime (15 years), such that only strong multi‐decadal trends will be anomalous and potentially affect populations.

Models projected changes in the rate of NPP in important regions visited by arctic terns towards the end of the 21st century (Figures 2 and 3). Strong declines exceeding 0.1 g C m^−2^ day^−1^ occur in the North Atlantic and along the west African coast, while increases of similar magnitude occurred in the Subantarctic Zone and the Weddell Sea where terns spend the boreal winter. These changes represent up to −30% of the seasonal means in NPP along the west African coast and in the Subantarctic, up to −50% in the North Atlantic and up to +100% (i.e. a doubling) of NPP in the Weddell Sea (Figure 2f,h). Weak changes occur elsewhere in the Southern Ocean where terns forage. A majority of models (9 of 12) agreed on the direction of changes in these key foraging regions (hatched areas in Figure 2b), providing some confidence in the projections for both moderate and extreme climate change scenarios, although we acknowledge that uncertainty remains (Tagliabue et al., 2021).

Changes in NPP become greater than the expected levels of variability in the North Atlantic foraging region, both when comparing the variability within an arctic terns' lifetime (*c*. 15 years, although some adults can live for over 30 years; Clapp et al., 1982) and relative to preindustrial conditions (Figure 3). According to the models, North Atlantic foraging conditions may have already become significantly worse in the early 21st century, or may yet arise in the coming decades. Whether poor foraging conditions emerge sooner or later depends on what is considered to be the envelope of normal conditions to which arctic terns are adapted (−1 to +1, grey shaded bar; Figure 3). An earlier emergence of anomalously poor foraging occurred when changes relative to the preindustrial (1850–1950) climate are considered (Figure 3a). Here it was assumed that the preindustrial climate conditions were optimum for arctic terns, and that there was no adaptive capacity to the new climates. A later emergence of poor foraging conditions occurred when the normal conditions for an arctic tern were those at the time of hatching (averaged over a 15 year lifespan; Figure 3b). Here the optimal conditions were considered to be those at the beginning of a tern's lifetime, and with strong declines in NPP over a 15‐year period, terns would encounter anomalously poor foraging conditions. This approach assumes strong adaption as every new generation is adapted to the average conditions at hatching. Thus, some generations of arctic terns were projected to encounter anomalously poor foraging in the North Atlantic with a high degree of confidence under the ‘fossil‐fuelled development’ scenario (SSP5‐8.5; solid black line in Figure 3b). Under the more moderate ‘middle‐of‐the‐road’ scenario (SSP2‐4.5), poorer foraging in the North Atlantic was less certain, and could depend on how quickly generations were able to adapt to new foraging conditions.

In contrast to the poor foraging conditions projected for the North Atlantic, there were weak or no notable projected changes in NPP in the other key foraging regions by 2100. While local changes in NPP were strong in these areas (Figure 2), when weighted by the area use of terns (Figure 1) they summed to weak trends. Consequently, it is uncertain whether future generations of terns will be affected by NPP changes off West Africa or in the Southern Ocean. Depending on normality assumptions and the climate change scenario, anomalously poor foraging may be encountered by arctic terns in the Benguela system (relative to preindustrial levels, SSP5‐8.5 particularly in 2100; Figure 3a), or not (within a terns' lifetime, neither SSP; Figure 3b). Similarly, anomalously rich foraging (i.e. increased NPP) could be encountered in the Southern Ocean (relative to preindustrial levels, both SSPs; Figure 3a), or not (within a terns' lifetime, neither SSP; Figure 3b). Trends in NPP at Amsterdam Island in the Subantarctic are sufficiently weak to not affect terns in either climate change scenario or given different assumptions of normality.

### 3.2 Sea ice

**FIGURE 4:**
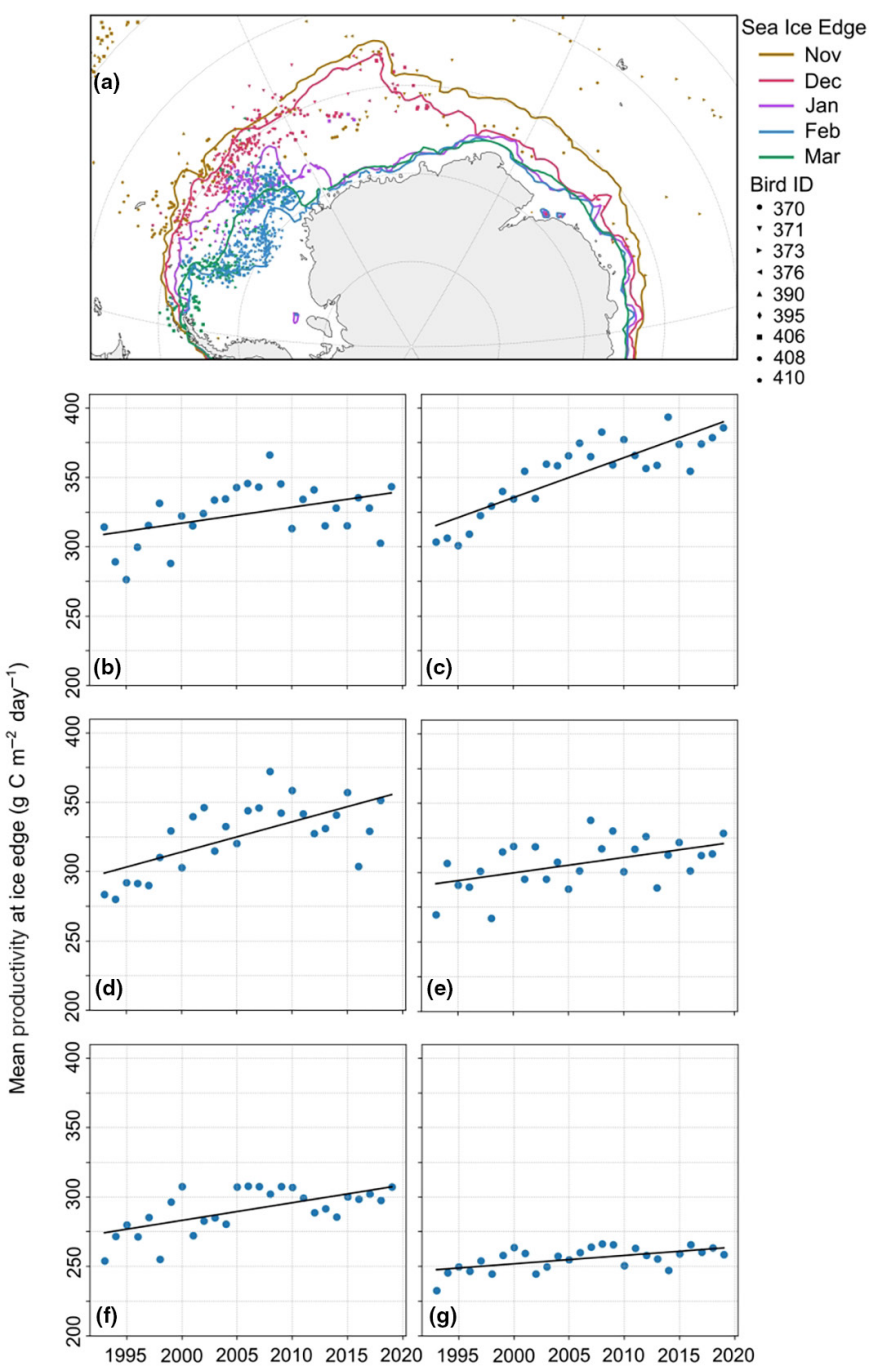
(a) Arctic tern locations recorded by geolocator devices during the austral summer of 2007–2008 (Egevang et al., 2010) at the observed sea ice edge. Changes in productivity at the observed Antarctic sea ice edge (from 60° W to 120° E) for (b) October, (c) November, (d) December, (e) January, (f) February and (g) March.

The sea ice edge is an important environmental variable that arctic terns associate with during the non‐breeding season in the Southern Ocean (Figure 4a; Redfern & Bevan, 2020). The modelled NPP from 1993 to 2019 was positively correlated with the sea ice extent, being greatest in November and December but lower from February to March, when arctic terns are in the Weddell Sea (Figure 4, Figure S3). NPP between 60° W and 120° E generally increased from 1993 to 2019, with the greatest increase during November and December (Figure 4).

**FIGURE 5:**
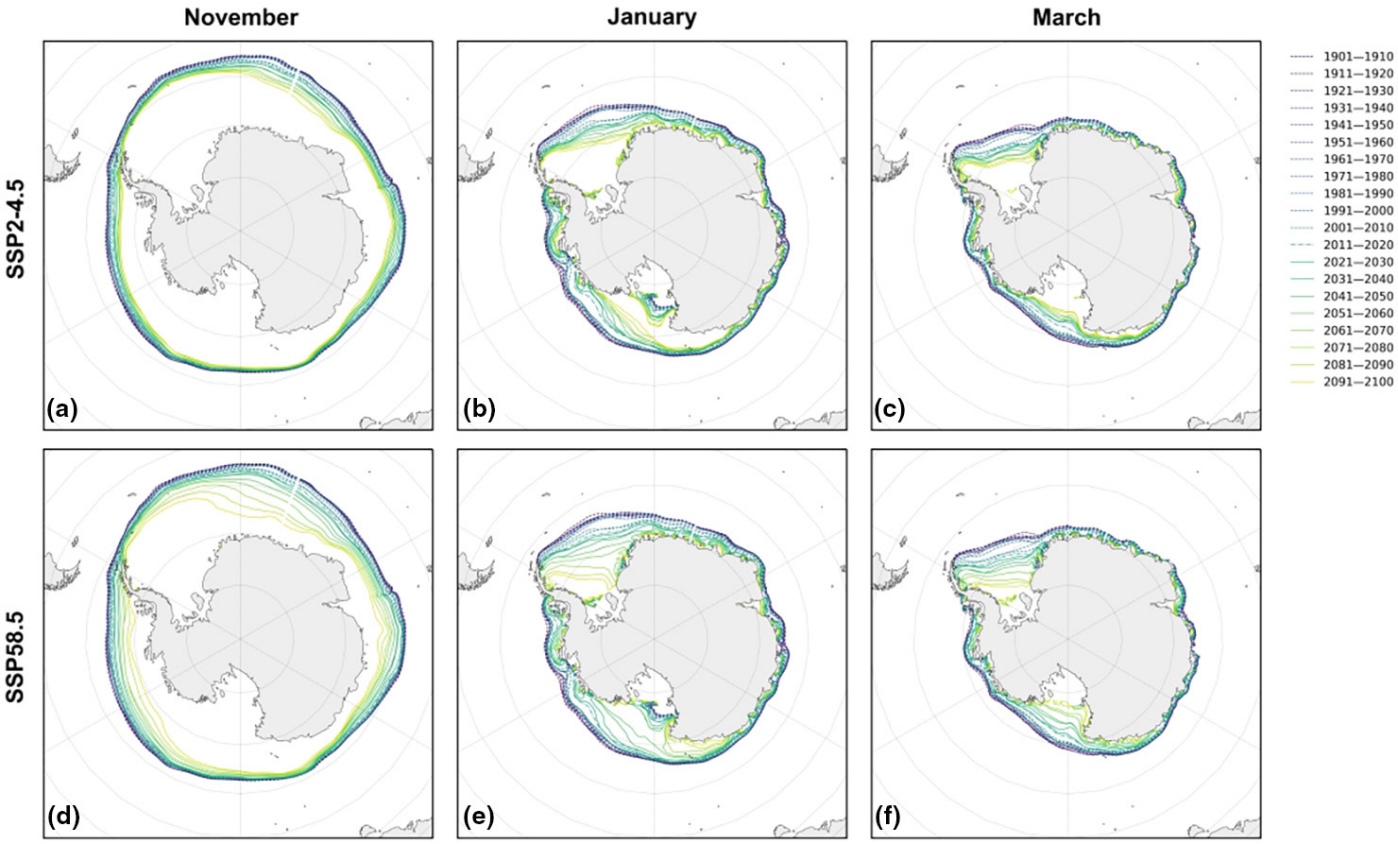
Historical (dashed lines) and projected (solid lines) sea ice edge for (a–c) climate scenario SSP2‐4.5 and (d–f) SSP5‐8.5 during the austral summer.

During the 6 months of the austral summer (October–March), the observed sea ice edge from 1981 to 2020 was greatest in October and November, which corresponded to the arrival of arctic terns. The ice extent was smallest and closer to the continent by February and March, although in the Weddell Sea, where arctic terns spend these months prior to their northbound migration, the sea ice extent was still quite large (Figure S4). The multi‐model decadal mean historical projections of sea ice closely resembled the observed extent, but incorrectly projected consistency (or in some areas a slight decrease) in the extent, whereas an increase was actually observed (Figure S5a,b). There was also a large spread between the models, with some overestimating and others underestimating the sea ice extent (Figure S5c). These large differences meant that any interpretation of future projections by these models should be treated cautiously. Nonetheless, all models agree that under both SSP scenarios there will be a decrease in summer sea ice extent by the end of the 21st century, and by March there are regions projected to have no sea ice coverage (Figure 5). However, the magnitude of change is hard to infer given that over recent decades models differed to observations and the causes are not fully understood.

### 3.3 Wind

**FIGURE 6:**
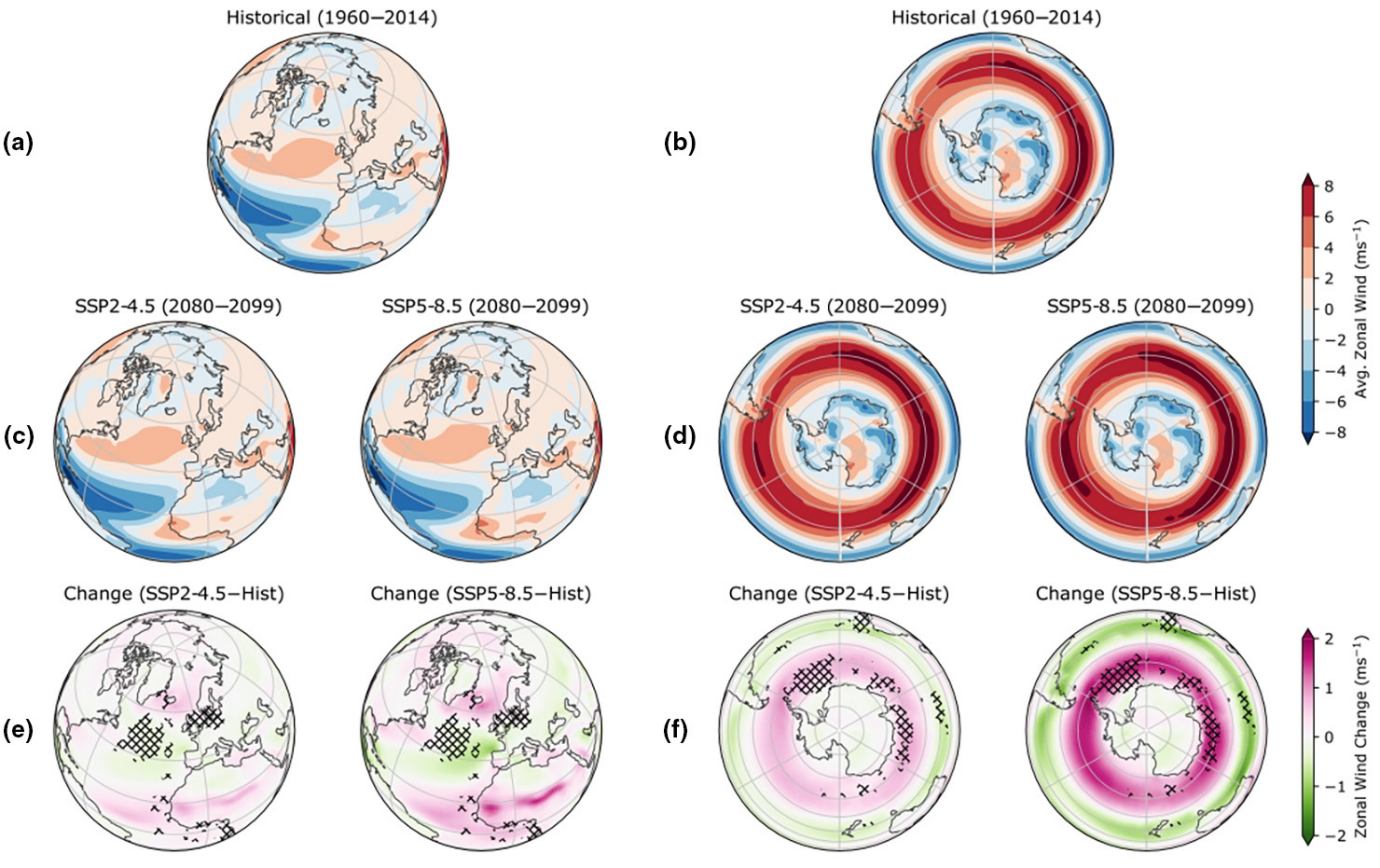
Projected changes to average zonal winds (along a constant latitude) in the northern hemisphere regions that arctic terns using the Atlantic flyway breed (left, from May to August) and Antarctic regions (right, November to March) in the two climate change scenarios (SSP2‐4.5 and SSP 5‐8.5). (a, b) The average historical zonal winds and (c, d) the projected zonal winds under the two scenarios. (e f) The corresponding relative changes to zonal winds between historical and future scenarios. The hatched regions represent areas where terns spend longer durations of time.

At high latitudes of the southern hemisphere (between 55° S and 65° S), where arctic terns spend the austral summer, projected westerly winds are likely to shift polewards and strengthen under both climate scenarios by approximately 1 and 2.5 m s^−1^ (SSP2‐4.5 and SSP5‐8.5 respectively; Figure 6). At 35° S–50° S, a corresponding weakening of westerly winds is projected by approximately 0.5 and 1.5 m s^−1^ (SSP2‐4.5 and SSP5‐8.5 respectively; Figure 6f). By contrast, at high latitudes of the northern hemisphere, across the circumpolar breeding range of arctic terns, there are minimal changes in zonal winds projected by 2100 (generally <1 m s^−1^) under both climate scenarios (Figure 6a,c,e).

**FIGURE 7:**
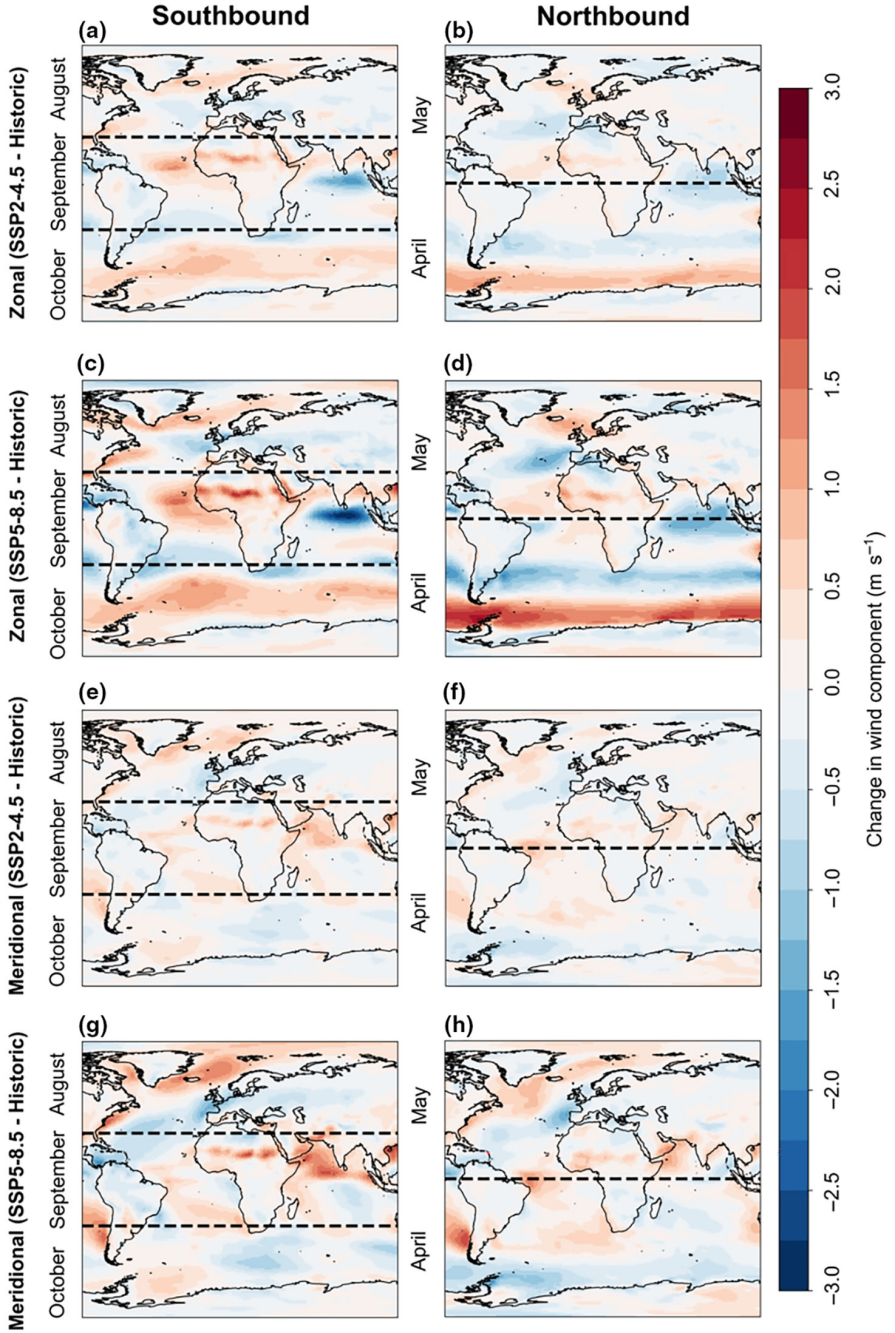
The projected changes of mean zonal and meridional winds compared to the historical conditions (1960–2014), with monthly averages corresponding with arctic tern presence during their southbound (left) and northbound (right) migrations. (a, b) Changes in zonal winds in the SSP2‐4.5 scenario. (c, d) Changes in zonal winds in the SSP5‐8.5 scenario. (e, f) Changes in meridional winds in the SSP2‐4.5 scenario. (g, h) Changes in meridional winds in the SSP5‐8.5 scenario.

During their southbound migration (August–October), the zonal and meridional winds experienced by arctic terns are projected to change by up to 1.5 and 3 m s^−1^ in the year 2100 compared to historical levels under the two climate scenarios SSP2‐4.5 and SSP5‐8.5 respectively (Figure 7). By comparison, the minimum power speed for arctic terns is approximately 6.8 m s^−1^ (Hedenström & Åkesson, 2016). There was also a notable projected eastward shift in the winds around Iceland, the Cape Verde Islands and in the southern Atlantic (Figure 7a,c). Historically, zonal winds (along a constant latitude) in the southern Atlantic were westerly and probably provided tailwind support for terns migrating below Southern Africa and into the Indian Ocean towards the key stopover region Amsterdam Island (Figure S6a). These westerlies are projected to increase by 2100. Meridional winds (along a constant longitude) around the Benguela region were historically likely headwinds for southbound migrating arctic terns, and are projected to increase by 0.5 and 1 m s^−1^ by 2100 (two climate scenarios SSP2‐4.5 and SSP5‐8.5 respectively; Figure S6a, Figure 7e,g).

The end‐of‐century wind projections during arctic tern northbound migration (April–May), show an intensification of easterlies close to the Antarctic continent of up to 2.5 m.s^−1^ (SSP5‐8.5), stronger westerlies below Africa, a slight strengthening of easterlies along the west coast of Africa, and a westward shift of 1 or 1.5 m.s^−1^ in the North Atlantic (SSP2‐4.5 and SSP5‐8.5 respectively; Figure 7b,d). Historically, southerly tailwinds were present along the coast of southern Africa from 35° S to 5° S (Figure S6b), but under both climate change scenarios there is a small weakening of southerly meridional winds by the end of the 21st century (Figure 7f,h).

**FIGURE 8:**
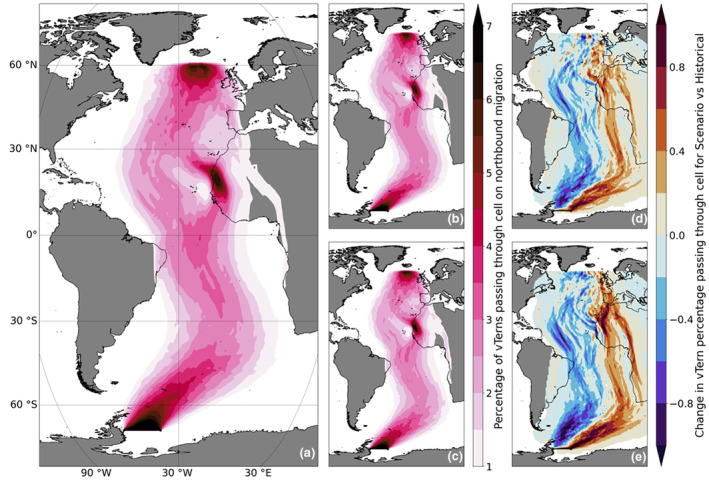
(a) The proportion of vTerns passing through each 1° cell in the simulated northward migration between 1950 and 1999 across all historical simulations, (b) the proportion of vTerns passing through each 1° cell in the simulated northward migration between 2050 and 2099 across all SSP2‐4.5, and (c) SSP5‐8.5 simulations. The percentage change in vTerns passing through each 1° cell from 1950–1999 to 2050–2099 across all (d) SSP2‐4.5 and (e) SSP5‐8.5 simulations. Cells with fewer than 1% of vTerns are not rendered for clarity.

The impact of winds were isolated on the simulated vTern northbound migrations, and as such the projected wind changes could indicate how future winds may impact actual arctic terns. Although the vTern northbound routes (from 1950 to 1999) did not replicate the fine‐scale movements of observed arctic tern migrations, vTern trajectories were sigmoidally shaped, but not to the same degree as tracked arctic terns (Figure 8; Alerstam et al., 2019; Egevang et al., 2010). vTern migration durations were similar (vTerns: 30.6 ± 4.1 days (mean ± SD; Figure S7); recorded arctic terns: 25–46 days; Alerstam et al., 2019; Egevang et al., 2010; Fijn et al., 2013), although they migrated over the African continent. The general pattern of vTern northbound routes did not differ between historical and future projected wind scenarios, but intensification of Southern mid‐latitude westerly winds caused a slight shift eastwards. This increased the proportion of vTerns crossing the African continent at least once from 50.3% in the historical simulations, to 55.3% and 56.9% under the SSP2‐4.5 and SSP5‐8.5 scenarios respectively (Figure 8). The projected migration duration did not change between the historical (30.6 ± 4.1 days) and either SSP2‐4.5 (30.3 ± 4.0 days) and SSP5‐8.5 (30.4 ± 3.9 days) future climate scenarios. However, some differences between vTern and observed arctic tern migration routes suggest that wind is not the only driver (a large proportion of tracked arctic terns fly close to the southwest coast of Africa (Figure 1a), but the majority of vTerns travelled through the central South Atlantic (Figure 8a); vTern trajectories also bifurcated over the subtropical North Atlantic and some followed the same western route over the central North Atlantic as observed arctic terns, but others deviated east along coastal North Africa; Figure S8).

## 4 DISCUSSION

Arctic terns rely on both polar regions, are long‐lived (up to 34 years; Clapp et al., 1982), and annually undertake the longest migration on the planet (Alerstam et al., 2019; Egevang et al., 2010; Fijn et al., 2013; Hromádková et al., 2020; McKnight et al., 2013; Volkov et al., 2017; Wong et al., 2021). Consequently, they are vulnerable to climate change, since polar regions are changing more rapidly than anywhere else on Earth (e.g. Previdi et al., 2021; Screen & Simmonds, 2010) and because climate‐driven trends and pressures are more apparent when assessed over broad geographical and temporal scales compared to local scales (Pörtner et al., 2022). Here we have assessed the projected changes to environmental conditions along a major flyway and in the Southern Ocean during the austral summer, and assessed the impact on arctic terns. The projected changes are likely to be relevant to a whole suite of species, such as birds that migrate along European–African flyways (BirdLife International, 2010a, 2010b; Breiner et al., 2022), marine vertebrates that use the same productive areas as arctic terns (Barendse et al., 2010; Davies, Carneiro, Tarzia, et al., 2021; De Broyer & Danis, 2011; Griffiths et al., 2011; Heerah et al., 2019), and animals that rely on sea ice as habitat for rest, foraging and reproduction (Massom & Stammerjohn, 2010).

### 4.1 Net primary production

Declines in NPP in the North Atlantic are likely (Bindoff et al., 2019), with projected declines of a large magnitude and strong inter‐model agreement. Intensifying stratification and slowing down of upper ocean overturning are likely to cause a reduction in both vertical and horizontal nutrient transport to the North Atlantic surface waters (Couespel et al., 2021; Whitt et al., 2019). Slower nutrient delivery shortens the growth season and limits primary production. The projected NPP declines in the North Atlantic therefore originate from physical, ‘bottom‐up’ changes to the ocean–atmosphere system, rather than biological feedbacks or ‘top‐down’ responses (i.e. grazing rates), which are considerably more uncertain in models (Laufkötter et al., 2015). The magnitude of these projected declines is, however, debatable. High‐resolution regional models report declines in North Atlantic NPP that are half as strong as those reported by lower resolution regional models, suggesting that the real ocean may experience more dampened trends than reported by the current suite of lower resolution Earth System Models (Couespel et al., 2021). Consequently, the signal to noise ratios reported here would be half as strong. However, it is noteworthy that even these dampened declines would still likely present as anomalously poor foraging conditions for arctic terns, albeit decades later, depending on the climate scenario, and on how rapidly new generations can acclimatise to new conditions.

In other key foraging regions (the Benguela upwelling system, Amsterdam Island in the Subantarctic Indian Ocean and the Southern Ocean), minimal changes in NPP are projected. Arctic terns likely experience dramatic variation in the productivity of the waters that they travel and feed over, so these projected changes in productivity are relatively minor in comparison. They may be able to mitigate against this heterogeneity by moving between sites, or by virtue of their long lives affording many breeding opportunities. Indeed, some bird species' are presently altering their behaviours to adapt to changing levels of prey availability. In response to decreases in productivity and poleward shifts of prey, the non‐breeding area of the planktivorous Antarctic prion (*Pachyptila desolata*, Gmelin, 1789) shifted southwards during the 20th century, while the broad‐billed prion (*P. vittata*, Forster, 1777) targeted a food‐rich seamount to mitigate the reduction in prey (Grecian et al., 2016). While this suggests an inherent resilience through the merit of a life lived at a large scale, there may be compounding effects of many small changes that integrate to impact arctic terns. Simultaneous declines at multiple foraging and stop‐over sites may mean that arctic terns may actually start to fail to find adequate food, even if NPP trends are weak when sites are assessed individually. Additionally, fishing stocks continue to be depleted, further exacerbating the situation. The collapse of the Namibian sardine fish stock in the latter half of the 20th century followed decades of fishery exploitation and consequently led to the near extinction of African penguins and Cape gannets in the region (90% and 95% reduction in populations respectively; Crawford, 2007). Antarctic krill commercial catches have increased to approximately 450,000 t of krill annually (Kawaguchi et al., 2009; Kawaguchi & Nicol, 2020; Trathan et al., 2022), and are major competition to seabirds that breed or overwinter in the Southern Ocean, including arctic terns (Trathan & Hill, 2016).

### 4.2 Sea ice

In contrast to the decreasing sea ice levels in the Arctic (Meier et al., 2014), from 1979 to 2017 there was a general increase in the Antarctic sea ice extent (Parkinson, 2019; Scott, 2023; Turner & Overland, 2009). Antarctic sea ice is, however, highly variable and the summer minimum in recent years was below average, reaching the lowest levels on record in 2023 (Scott, 2023). The precise mechanisms behind these trends are not fully understood, although many are proposed (Armour et al., 2016; Ferreira et al., 2015; Holland & Kwok, 2012). Despite their inability to reproduce trends over the past few decades, all models agree that a decrease in sea ice extent is likely by the end of the 21st century. Furthermore, there have also been small increases in production by sea ice algae over the last 30 years, perhaps due to increased light penetration of the thinning ice (Pinkerton & Hayward, 2021), that align with projections of rising productivity in future decades (Bindoff et al., 2019). The effect of potential losses in sea ice and gains in NPP on arctic terns is highly uncertain, not only because of the due caution in interpreting Antarctic sea ice extent using Earth System Models (Roach et al., 2020; Shu et al., 2020), but also because krill require sea ice habitat (Siniff et al., 2008), but rely on phytoplankton for foraging. Future trends in these key environmental variables act in opposing ways on krill stocks, and by extension in opposing ways for foraging arctic terns during their overwintering period in Antarctica.

Responses to changes in Antarctic sea ice by other marine megafauna are variable. For example, in ice‐free breeding seasons, Adélie penguins (*Pygoscelis adeliae*, Hombron & Jacquinot, 1841) travelled more rapidly and efficiently by swimming rather than walking, meaning they could search for prey over larger areas (Watanabe et al., 2020), while crabeater seal (*Lobodon carcinophaga*, Hombron and Jacquinot, 1842) pups suffer greater mortality as they need to remain on large ice floes, and adults have less access to krill, which decreases along with the sea ice extent (Siniff et al., 2008). Adult Emperor penguins (*Aptenodytes forsteri*, Gray, 1844) survive better with greater sea ice area, but their chicks have reduced hatching success (Barbraud & Weimerskirch, 2001). Given the complexities in projecting sea ice areas by 2100, it is still challenging to unravel how species may respond, though animals like arctic terns, that are not restricted as central place foragers, can seek alternative foraging locations (Redfern & Bevan, 2020) and may be better able to cope with changes compared to many marine top predators that breed during the austral summer (Ichii et al., 2007).

### 4.3 Winds

Here we report minimal projected changes to the zonal and meridional winds during southbound and northbound arctic tern migrations under both climate scenarios. However, we do identify some areas where arctic terns may have to compensate for wind shifts. Stronger easterly crosswinds around the northwest African coast and stronger southerly winds around southern Africa may create stronger headwinds during the southbound migration, but stronger tailwinds during the northbound migration. Such compensation is likely to be more energetically costly. However, arctic terns have coped with changing winds before. The strong westerlies in the Southern Ocean shifted equatorward during the Little Ice Age (*c*. 13th–19th centuries) as temperatures decreased (Perren et al., 2020).

Arctic tern foraging is currently unlikely to be impacted by prevailing wind conditions during the breeding season in the northern hemisphere (Morten et al., 2022), and data in the present study suggest it is unlikely that this will change by the end of the century. However, in the Southern Ocean there are likely to be changes in zonal winds (i.e. the westerlies), which may be important because over the course of the austral summer, foraging arctic terns tend to move westwards towards the Weddell Sea, and may be aided by easterly prevailing winds near the Antarctic coast. These winds are projected to weaken and contract towards the coast under both climate scenarios, which may impact their flight efficiency. Other species, such as albatross, that primarily move throughout the Southern Ocean using soaring flight (Sachs et al., 2013), may need to alter their flight paths to cope.

In the present study, vTerns allowed us to test the parameters that are likely to be important to the overall route and timing of arctic tern migration. Despite our simple behavioural assumptions of the simulated northbound migration (i.e. passive advection with surface winds and a constant airspeed directed towards Iceland during daylight hours), trajectories of vTerns generally resembled observed arctic tern northbound migration routes. It thus plausible that the sigmoidal northwards migration route of arctic terns could be driven by surface winds, rather than active flight (see also Hromádková et al., 2020). Of course, this may vary at smaller spatial and temporal scales (e.g. in the present study, wind gusts would have been smoothed out by daily means obtained from relatively coarse spatial grids ≥1°) by terns moving away from detrimental winds, and with movements towards favourable foraging opportunities en route. Indeed, while the duration of migration was similar, real arctic terns spend on average 10 days longer migrating north than our vTerns predicted (Alerstam et al., 2019; Egevang et al., 2010; Fijn et al., 2013), which may be due to energetically costly deviations from the most direct routes towards better foraging opportunities, and/or avoiding poor wind conditions. Additionally, vTerns flew at consistent airspeeds for 14 h per day, this is unlikely for actual arctic terns, particularly when they are fishing (Hedenström & Åkesson, 2016).

Under both climate scenarios, simulated vTerns shifted eastwards in the South Atlantic due to the projected intensification of westerly winds in the Subantarctic. However, the shift was largely imperceptible, involving changes in the number of terns migrating through more eastern corridor by less than 1%. Furthermore, the response of real migrating arctic terns may be different than that demonstrated by vTerns. While arctic terns have been recorded migrating overland (Redfern & Bevan, 2019), they would be unable to feed on their usual marine prey during the trans‐African portion of their northbound migration. It is therefore much more likely that terns would continue to migrate over water irrespective of novel wind conditions, such as the stronger westerlies and likely expend more energy in the future doing so. If they also experience less tailwind support by 2100, migration may be slower and incur additional energetic costs in the future, when it becomes less energetically favourable to remain over the ocean. Overall, however, we find limited impacts of wind changes on arctic tern migration.

### 4.4 Conclusions

The impacts of projected changes to NPP, sea ice and winds by 2100 on arctic terns are likely to be minimal, although the cumulative effects of these changes are unclear. Presently, declines in some arctic tern populations are reported (BirdLife International, 2018), both in the size of nesting colonies, and in breeding success (Burnham et al., 2017; Henri et al., 2020; Maftei et al., 2015; Petersen et al., 2020; Vigfúsdóttir et al., 2013), but the future effects of environmental change on arctic tern populations remain to be seen. Although the variables projected here are unlikely to impact arctic terns during their migration or non‐breeding seasons, the cumulative effect of many small changes to the environment (including metrics we did not investigate such as resource extraction by fisheries) are uncertain, and the huge spatial distribution of colonies and long generation times are likely to create a lag in demographic changes.

Arctic terns are unique in their reliance on profitable environmental conditions at both polar regions of the Earth every year. Their breeding failure in the Arctic in particular may reflect changing conditions on arrival, or subtle reductions in the resources they can carry with them from Antarctica at the end of the austral summer, or a reduction in favourable conditions en route. Arctic terns, and many other species that breed at high latitudes, have a limited capacity to shift breeding sites further northwards to follow climate envelopes (Convey & Peck, 2019). Mitigating the impacts of climate change on migratory species, particularly those that rely on multiple different sites under different jurisdictions, is challenging and requires a holistic approach (Conners et al., 2022; Gilmour et al., 2022). Improved management and policy changes could also minimise other exacerbating factors, such as predation by invasive species (Wanless et al., 2007). Large marine protected areas (MPAs), such as the recently designated North Atlantic Current and Evlanov Seamount could relieve some pressures for millions of seabirds (Davies, Carneiro, Campos, et al., 2021; Davies, Carneiro, Tarzia, et al., 2021) and a suite of other taxa including sharks (Queiroz et al., 2016) and cetaceans (Pike et al., 2020). Ultimately, as climate change progresses and key carbon emission targets are missed (Hale et al., 2022), dramatic changes to emissions and policy are key to permit species like arctic terns the space to keep adapting.

## FUNDING INFORMATION

J.M.M. and N.C.W. were supported by NERC GW4+ Doctoral Training Partnership studentships from the Natural Environment Research Council (NE/L002434/1). P.J.B. was supported by the ARISE project (NE/P006035/1), part of the Changing Arctic Ocean programme, jointly funded by the UKRI Natural Environmental Research Council (NERC) and the German Federal Ministry of Education and Research (BMBF). I.A.G. was supported by a University of Bristol PGR scholarship. DAW was supported by a STFC studentship from the Science and Technology Facilities Council (ST/V506667/1). Tracking data obtained by authors were funded by a National Geographic grant (WW1‐286R‐18) awarded to L.A.H.

## CONFLICT OF INTEREST STATEMENT

The authors declare no conflict of interest.

## Supporting information


Data S1.

## Data Availability

*Tracking data*: The tracking data that were collected and support the findings of this study are available in the Seabird Tracking Database at 2356146398 https://data.seabirdtracking.org/dataset, reference number 1905. Additional tracking data that support the findings of this study are openly available in Dryad at https://doi.org/10.5061/dryad.d6080nt and available upon request at https://data.seabirdtracking.org/dataset/739. *Environmental variables*: The data that support the findings of this study are openly available in JASMIN at https://jasmin.ac.uk/. All CMIP6 model output is freely available on the Earth System Grid Federation (https://esgf.llnl.gov/). Global ocean biogeochemistry hindcast simulations are available on the Copernicus Marine Database (https://resources.marine.copernicus.eu/).
