# Supplementary material for: Global warming and arctic terns: Estimating climate change impacts on the world's longest migration
Source: Glob Chang Biol. 2023 Jul 26;29(19):5596–614. doi: 10.1111/gcb.16891 (PMC10946559; doi:10.1111/gcb.16891)
Supplement: Supplementary file 1 — Data S1. [file GCB-29-5596-s001.docx]

**SUPPLEMENTARY FIGURES**

**
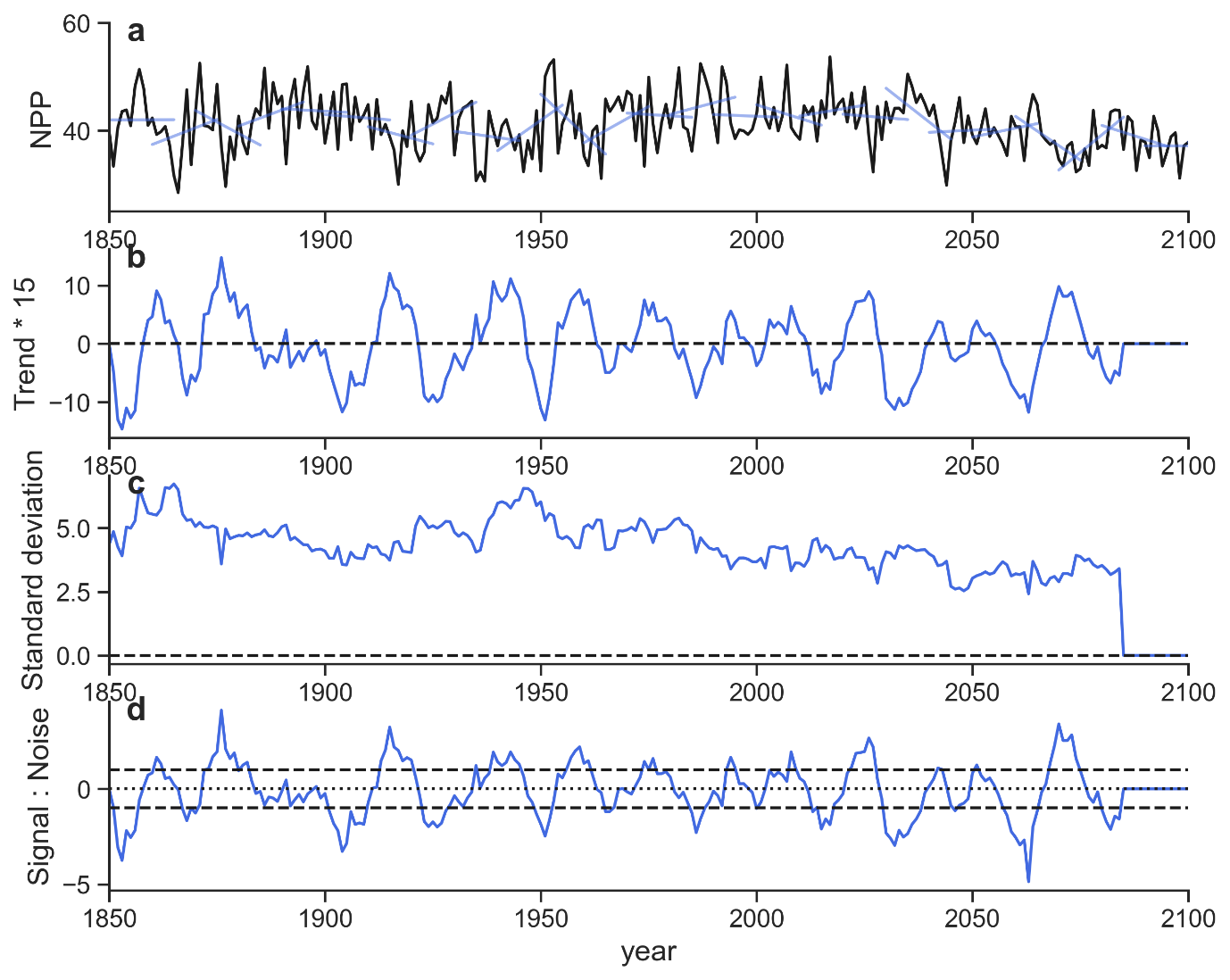
Supplementary Figure 1**. An example of how to calculate the signal to noise ratio assuming that birds of each successive generation are able to adapt to the background climate conditions they are born into. (**a**) the multi-model mean net primary productivity (NPP) rates in grams C m^-2^ year^-1^ at 48°N and 50°W (black) and the 15-year linear trends in NPP at this location each year from 1850-2100 (blue lines). (**b**) The 15-year linear trends at each consecutive year, which represents the trends seen by each consecutive generation of Arctic terns assuming a lifetime of 15 years. (**c**) The standard deviation of each consecutive 15-year period. (**d**) The signal to noise ratio at each consecutive year, which is the 15-year linear trend divided by the 15-year standard deviation (values in panel b divided by values in panel c). Note that the trend, standard deviation and signal to noise ratio is zero after 2085 because the model projections of NPP end at 2100.


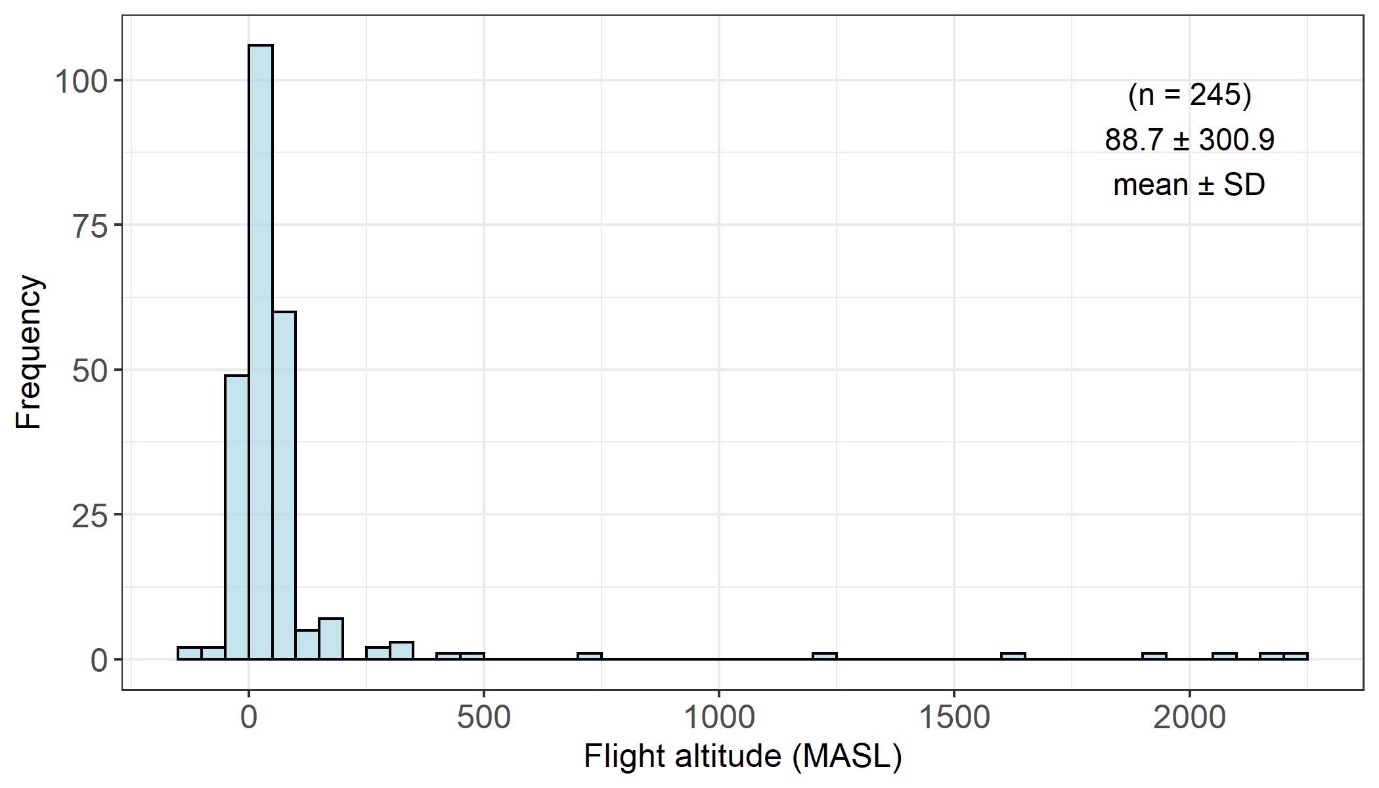


**Supplementary Figure 2.** The flight altitude recorded during the southbound migration of two arctic terns carrying GPS tracking devices.

**
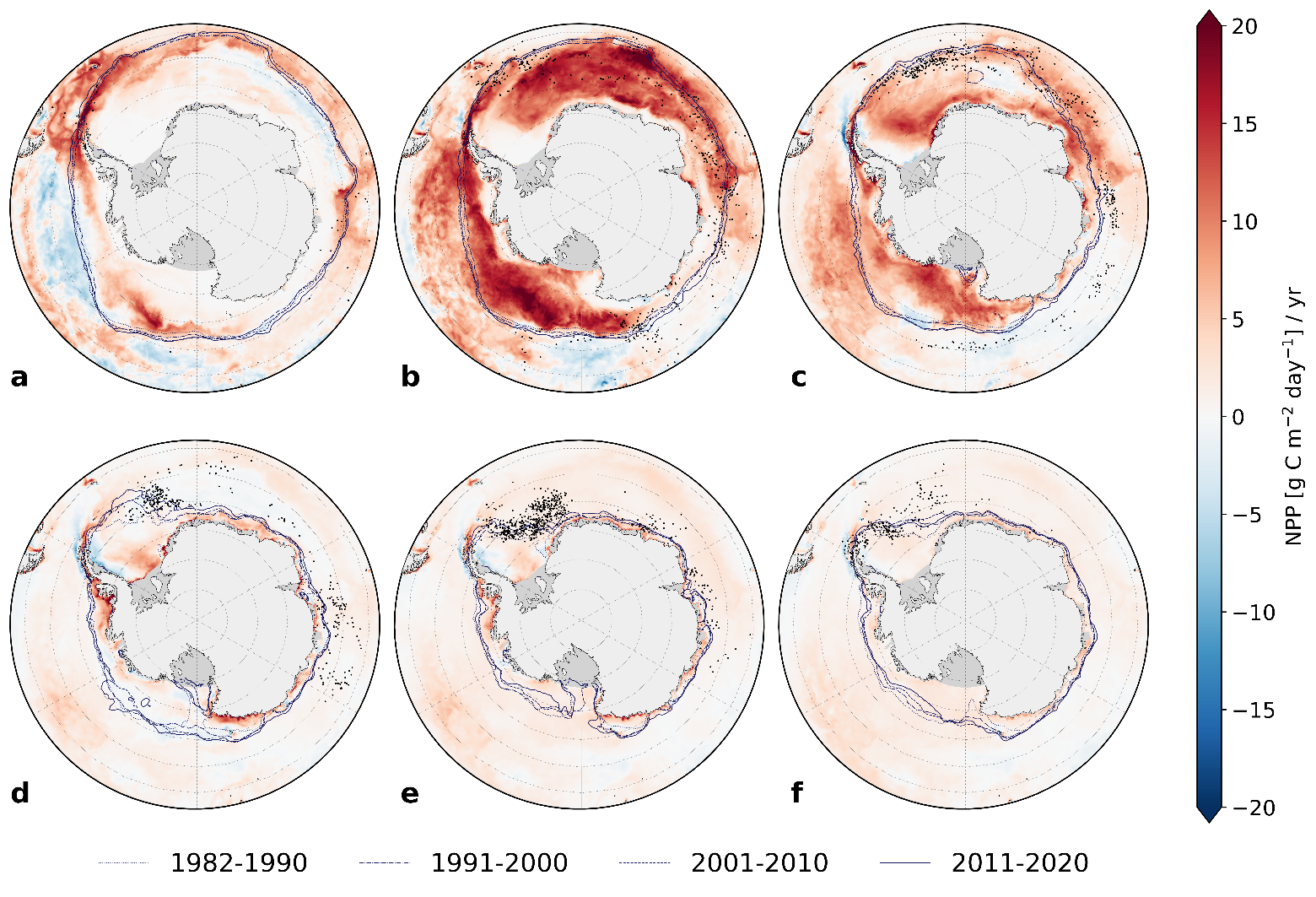
**

**Supplementary Figure 3**. The monthly trend in observed net primary productivity during (**a – f**) October to March from 1993 – 2019. Lines are the decadal mean observed sea ice edge (from 1982 **–** 2020) and points are recorded arctic tern locations collected from 2007 – 2019.


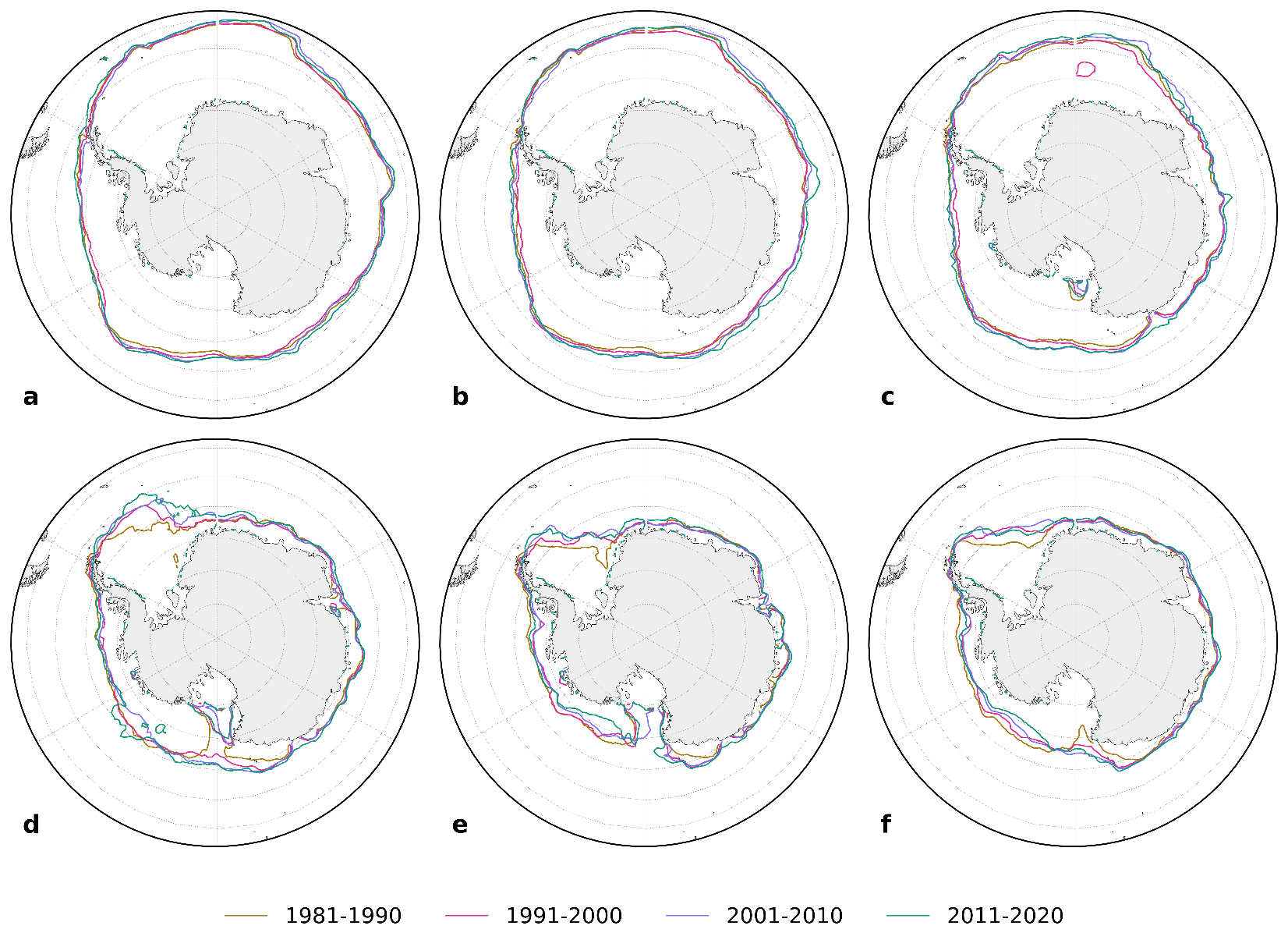

**Supplementary Figure 4**: Observed sea ice edge (decadal mean) for every austral summer month (1982-2020). (**a**) October, (**b**) November, (**c**) December, (**d**) January, (**e**) February, (**f**) March.


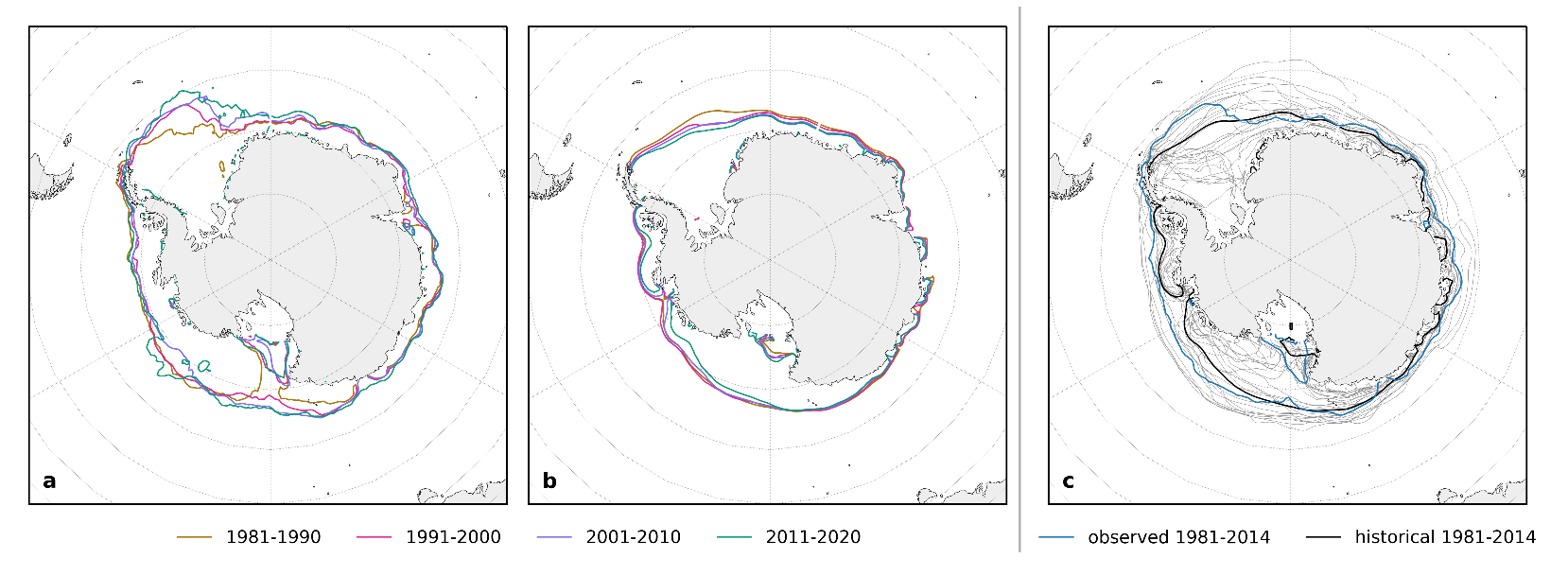

**Supplementary Figure 5:** A comparison between the average decadal sea ice edge during January that was (**a**) observed and (**b**) historically projected by CMIP6 multi-model means. (**c**) The observed and modelled historical sea ice edge averaged from 1981 - 2014. The historically projected sea ice edges from each CMIP6 model used to calculate the multi-model mean (Table 1) are shown in grey and indicate the spread of model results.


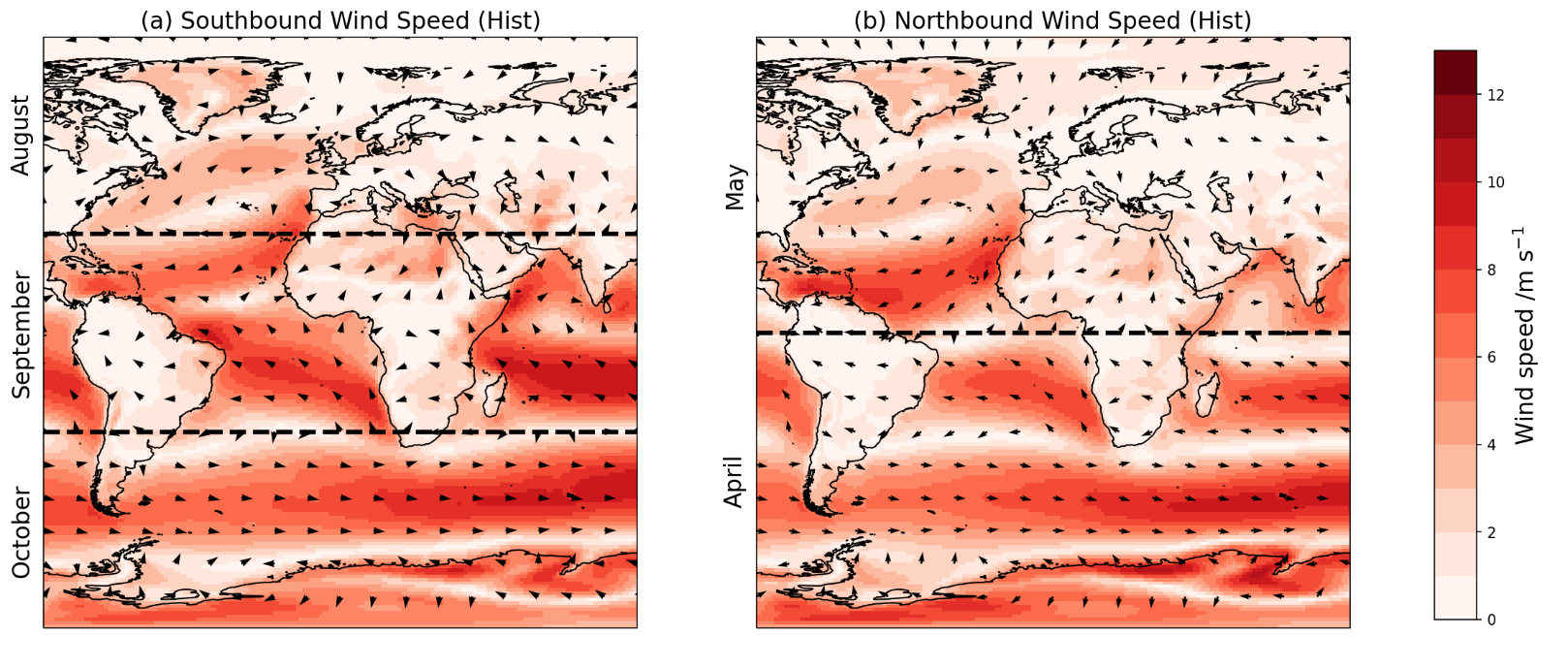

**Supplementary Figure 6:** Multimodel mean wind speed from historical simulations (1960 - 2014) during southbound **(a)** and northbound **(b)** migration months. Arrows denote the direction of the winds.


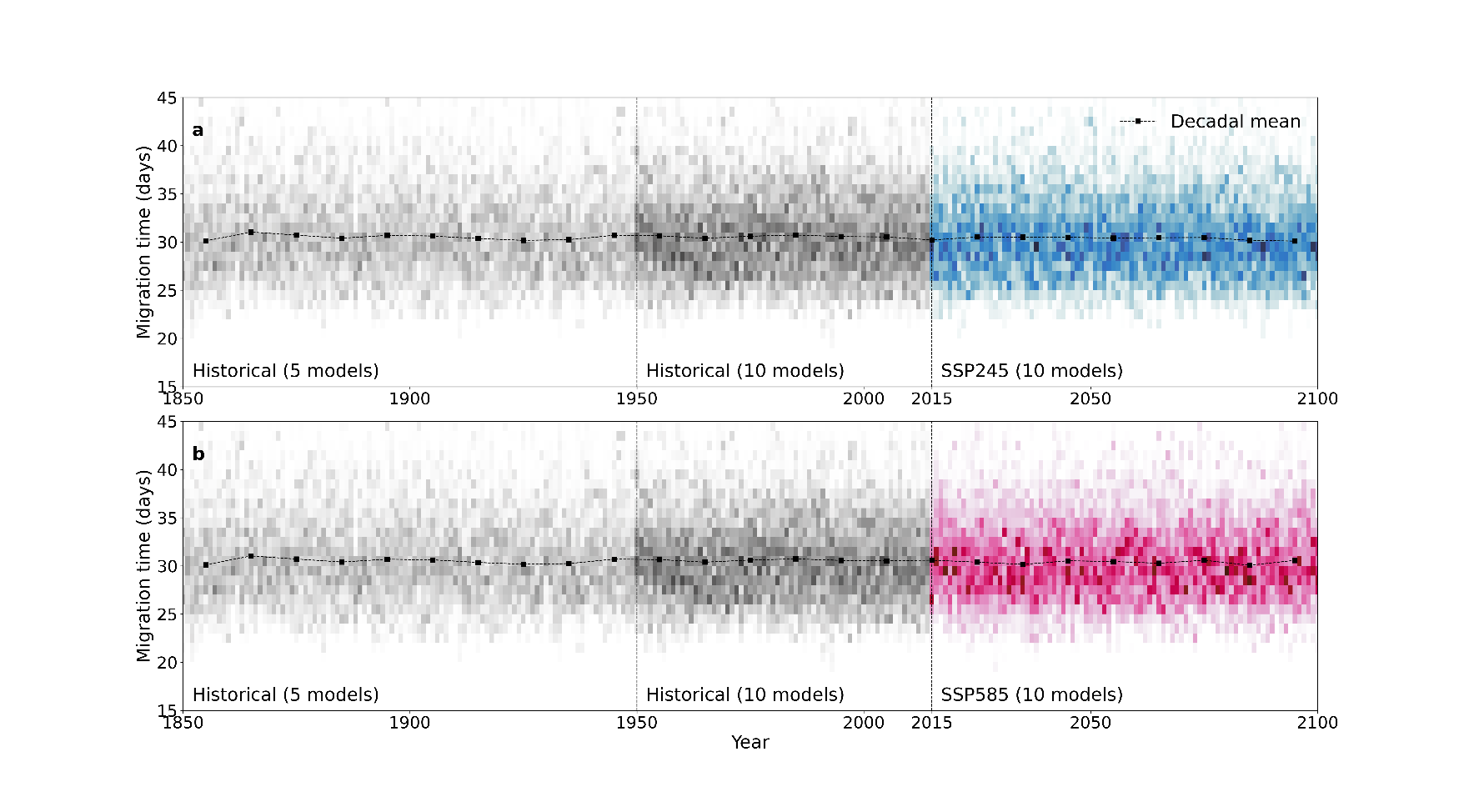
**Supplementary Figure 7**: Histogram of time taken for the northbound vTern migration across all (**a**) Historical+SSP2-4.5  and (**b**) Historical+SSP5-8.5 simulations. Only five of the ten models used in these simulations included data for pre-1950. The time taken for each individual vTern trajectory was binned by year (x-axis) and migration time in days (y-axis), with the saturation representing the number of vTern trajectories per bin. Markers and dotted lines represent the mean vTern migration time per decade. The mean migration time over the historical period (1950 – 1999) is 30.6 ± 4.1 days, compared to 30.3 ± 4.0 and 30.4 ± 3.9 days for SSP2-4.5 and SSP5-8.5 respectively (2050 – 2099 in both cases). There is no significant trend in annual mean northbound migration time over the assessed periods.


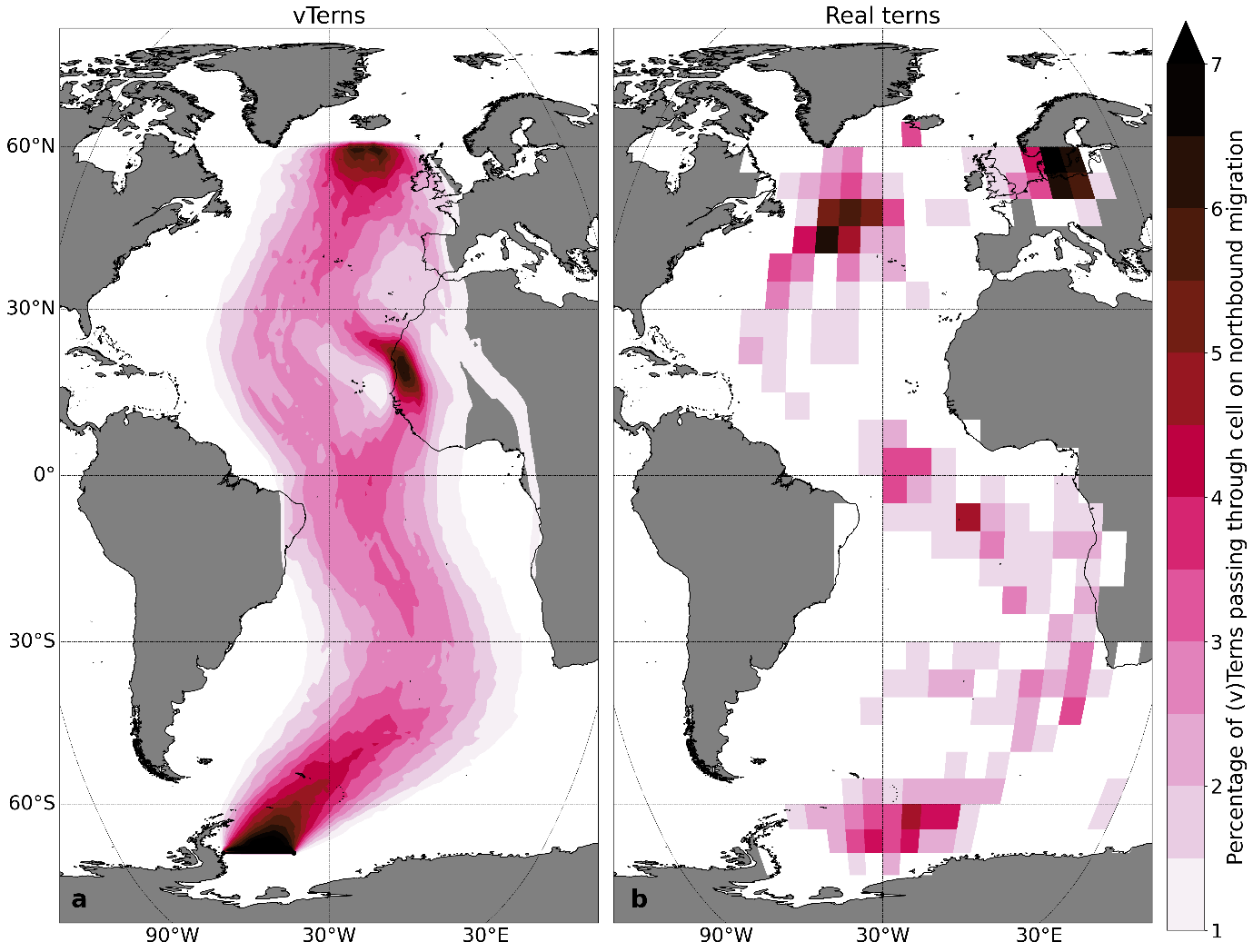


**Supplementary Figure 8**: Comparison of (**a**) northbound vTern trajectories from Historical simulations (see Figure 8(a), main text), and (**b**) observed arctic tern migration paths from GPS (this study) and geolocator (from: Egevang et al. 2010, Alerstam et al. 2019) tracking data (gridded to a 5° grid), from March to June.
